# Changes in active-site geometry on X-ray photoreduction of a lytic polysaccharide monooxygenase active-site copper and saccharide binding

**DOI:** 10.1107/S2052252522007175

**Published:** 2022-08-17

**Authors:** Tobias Tandrup, Sebastian J. Muderspach, Sanchari Banerjee, Gianluca Santoni, Johan Ø. Ipsen, Cristina Hernández-Rollán, Morten H. H. Nørholm, Katja S. Johansen, Flora Meilleur, Leila Lo Leggio

**Affiliations:** a Department of Chemistry, University of Copenhagen, Universitetsparken 5, 2100-DK, Copenhagen, Denmark; b ESRF, Structural Biology Group, 71 avenue des Martyrs, 38027 Grenoble cedex, France; c Department of Geosciences and Natural Resource Management, University of Copenhagen, 1958-DK, Frederiksberg, Denmark; d Novo Nordisk Foundation Center for Biosustainability, Technical University of Denmark, 2800-DK, Kgs. Lyngby, Denmark; e Department of Molecular and Structural Biochemistry, North Carolina State University, Campus Box 7622, Raleigh, NC 27695, USA; f Neutron Scattering Division, Oak Ridge National Laboratory, PO Box 2008, Oak Ridge, TN 37831, USA; University of Chicago, USA

**Keywords:** active-site geometry, X-ray photoreduction, lytic polysaccharide monooxygenases, LPMO, *Lentinus similis*, *Thermoascus aurantiacus*, active-site copper, saccharide binding

## Abstract

The recently discovered lytic polysaccharide monooxygenases (LPMOs) are Cu-containing enzymes capable of degrading polysaccharide substrates oxidatively. The generally accepted first step in the LPMO reaction is the reduction of the active-site metal ion from Cu^2+^ to Cu^+^. Here we have used a systematic diffraction data collection method to monitor structural changes in two AA9 LPMOs, one from *Lentinus similis* (*Ls*AA9_A) and one from *Thermoascus auranti­acus* (*Ta*AA9_A), as the active-site Cu is photoreduced in the X-ray beam. For *Ls*AA9_A, the protein produced in two different recombinant systems was crystallized to probe the effect of post-translational modifications and different crystallization conditions on the active site and metal photoreduction. We can recommend that crystallographic studies of AA9 LPMOs wishing to address the Cu^2+^ form use a total X-ray dose below 3 × 10^4^ Gy, while the Cu^+^ form can be attained using 1 × 10^6^ Gy. In all cases, we observe the transition from a hexa­coordinated Cu site with two solvent-facing ligands to a T-shaped geometry with no exogenous ligands, and a clear increase of the θ_2_ parameter and a decrease of the θ_3_ parameter by averages of 9.2° and 8.4°, respectively, but also a slight increase in θ_T_. Thus, the θ_2_ and θ_3_ parameters are helpful diagnostics for the oxidation state of the metal in a His-brace protein. On binding of cello-oligosaccharides to *Ls*AA9_A, regardless of the production source, the θ_T_ parameter increases, making the Cu site less planar, while the active-site Tyr—Cu distance decreases reproducibly for the Cu^2+^ form. Thus, the θ_T_ increase found on copper reduction may bring *Ls*AA9_A closer to an oligosaccharide-bound state and contribute to the observed higher affinity of reduced *Ls*AA9_A for cellulosic substrates.

## Abstract 2

The recently discovered lytic polysaccharide monooxygenases (LPMOs) are Cu-containing enzymes capable of degrading polysaccharide substrates oxidatively. Here we have monitored the Cu-site geometry in two AA9 LPMOs, one from *Lentinus similis* (*Ls*AA9_A) and one from *Thermoascus auranti­acus* (*Ta*AA9_A), as the active-site Cu is photoreduced in the X-ray beam. In addition, we have characterized in detail the structural effects of oligosaccharide binding in differently photoreduced states of *Ls*AA9_A.

## 1. Introduction

25–50% of all proteins contain metal cofactors (metallo­proteins) (Hoppert, 2011 ▸; Waldron *et al.*, 2009 ▸) and govern a range of biological functions, such as non-enzymatic electron charge transfer, metal transport and storage (Hoppert, 2011 ▸), and catalysis (Chen *et al.*, 2019 ▸). Redox activities, specifically, are often associated with enzymes containing iron and copper (Hoppert, 2011 ▸; Bowman *et al.*, 2016 ▸). Protein Cu centres are involved in metal transport, regulation and oxidation of various com­pounds (Festa & Thiele, 2011 ▸), and are often defined by the number and character of ligands coordinating the metal (Solomon *et al.*, 2014 ▸).

In X-ray crystallography experiments, crystals of metalloproteins absorb energies in the X-ray range more readily than a de-metallized form of the protein due to the presence of the transition metals (Handing *et al.*, 2018 ▸). Metalloproteins are therefore more predisposed to radiation damage than proteins not containing metals. With the increase in flux and intensity of X-rays at the new synchrotron beamlines, X-ray studies on metalloproteins become more challenging. It is estimated that only 10% of the inter­acting X-ray photons are scattered by crystals during data collection (Bowman *et al.*, 2016 ▸). The inter­acting but non-scattering photons may be absorbed and result in either heat increase or radiation damage (Helliwell, 1984 ▸). Photoreduction is therefore commonly observed in the structures of metalloproteins determined by X-rays. Metal centres may be found to change electronically and structurally (Bowman *et al.*, 2016 ▸), as can be monitored by spectroscopy and/or shown by increasing bond distances and altered bond angles between the metal and its ligands (Bowman *et al.*, 2016 ▸; Frankaer *et al.*, 2014 ▸; Corbett *et al.*, 2007 ▸; Antonyuk & Hough, 2011 ▸; Yano *et al.*, 2005 ▸). Such distorted metal coordination geometry may lead to the incorrect identification of the metal ion and the catalytic mechanism, especially of a redox protein.

When performing a diffraction experiment, the incoming photons of the X-ray beam may reduce the metal of metalloproteins even at low doses. This makes the structure determination of the native/resting state difficult. One way of circumventing photoreduction is to use neutrons instead of X-rays for crystallographic studies of metalloproteins (Schröder & Meilleur, 2021 ▸), though this imposes severe limitations on the systems that can be studied, as much bigger crystals are required for neutron diffraction. Furthermore, reduction of transition metals by X-rays may be a convenient reaction trigger for studies of enzymatic reactions in crystals (Bourgeois & Weik, 2009 ▸; Bourgeois & Royant, 2005 ▸). Two strategies used to study reaction inter­mediates structurally are either a real-time approach (serial crystallography) or a trapping approach (conventional crystallography) (Bourgeois, 2017 ▸; Schotte *et al.*, 2012 ▸).

**Figure 1:**
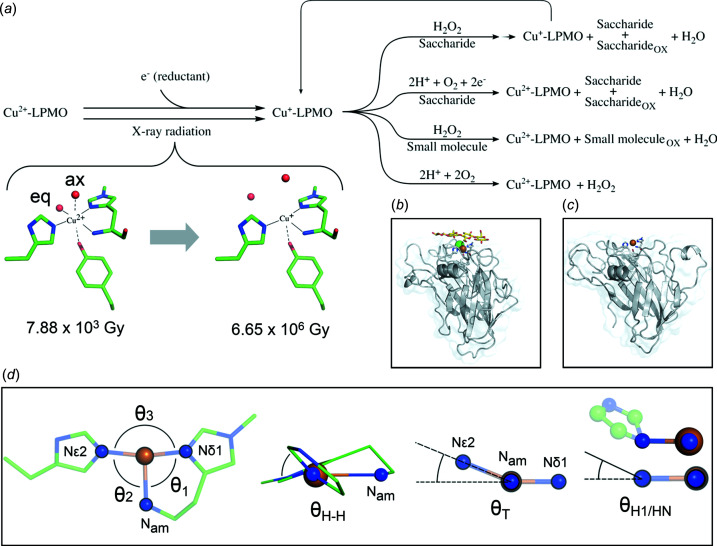
Illustrated is a simplified lytic polysaccharide monooxygenase (LPMO) reaction scheme. (*a*) LPMOs can engage in a variety of reactions depending on the specific enzyme. After Cu reduction, reactions with O_2_ and/or H_2_O_2_ may occur, involving saccharides or a variety of small mol­ecules, in some cases in several steps (Brander, Tokin *et al.*, 2021 ▸; Brander, Lausten *et al.*, 2021 ▸). Saccharide substrates produce smaller oxidized and/or non-oxidized saccharides as products. The first step in the reaction can be triggered either chemically by a reducing agent or using X-ray photons. The transition from Cu^2+^ to Cu^+^ results in expulsion of the equatorial (eq) and axial (ax) ligand water mol­ecules in the type II Cu site. These ligands may be different based on the crystallization liquor com­ponents. Upon binding of the substrate, the axial water mol­ecule is displaced. The *Ls*AA9_A His-brace is shown at low (left; PDB entry 7pxi) and high (right; PDB entry 7pxn) X-ray dose (see results). (*b*) The overall structure of *Ls*AA9_A binding cello­tetra­ose (PDB entry 6ydg). (*c*) The overall structure of *Ta*AA9_A (PDB entry 3zud). (*d*) The bond angles referenced throughout the article are defined in Vu & Ngo (2018 ▸). Angles θ_1_, θ_2_ and θ_3_ are measured from N—Cu—N as indicated. θ_H–H_ is the angle between the two His imidazole ring planes. θ_T_ is the angle between the N^δ1^—Cu—N_am_ plane and the N^ɛ2^—Cu line. θ_H1_ (also denoted θ_HN_) is the angle between one His imidazole ring plane and the line from Cu to the Cu-inter­acting N atom of the same imidazole ring.

Lytic polysaccharide monooxygenases [LPMOs, reviewed in Tandrup *et al.* (2018 ▸), Vu & Ngo (2018 ▸), Wang *et al.* (2020 ▸) and Eijsink *et al.* (2019 ▸)] were discovered just over 10 years ago and are metalloenzymes containing a type II Cu centre (Quinlan *et al.*, 2011 ▸). A Cu^2+^ ion is coordinated by two histidines in a motif known as the histidine brace (His-brace), where the N-terminal His is often N^ɛ2^-methyl­ated in LPMOs of fungal origin and coordinates Cu using both the N-terminal nitro­gen and the N^δ1^ atom (see Fig. 1 ▸) (Quinlan *et al.*, 2011 ▸). The His-brace motif is also found in other non-LPMO copper proteins (Labourel *et al.*, 2020 ▸; Garcia-Santamarina *et al.*, 2020 ▸; Udagedara *et al.*, 2019 ▸; Cao *et al.*, 2018 ▸). Known LPMOs degrade lignocellulose-, hemicellulose-, chitin-, starch- or pectin-containing biomass oxidatively, and have a boosting effect on saccharification together with glycoside hydro­lases (Harris *et al.*, 2010 ▸; Lo Leggio *et al.*, 2015 ▸; Quinlan *et al.*, 2011 ▸; Vaaje-Kolstad *et al.*, 2010 ▸; Zerva *et al.*, 2020 ▸), thus attracting considerable industrial inter­est for the production of biofuel (Hemsworth *et al.*, 2013 ▸; Müller *et al.*, 2015 ▸; Beeson *et al.*, 2015 ▸; Johansen, 2016 ▸) and other biotechnological applications (Ipsen *et al.*, 2021 ▸). LPMOs are widespread in nature and have been identified in all domains of life, though so far not in mammals. In the CAZy (Carbohydrate-Active enZymes) database, they are classified as Auxiliary Activity families 9–17 (family 12 excluded) (Lombard *et al.*, 2014 ▸), with AA17 recently discovered in oomycetes having activity against pectin (Sabbadin *et al.*, 2021 ▸).

LPMOs cleave the glycosidic bond, producing both oxidized (C1 and/or C4) and non-oxidized chain ends. Their mechanism (see the simplified LPMO reaction scheme in Fig. 1 ▸) has been researched extensively since their discovery. An essential (though probably not rate limiting) step is the reduction of the metal cofactor Cu^2+^ to Cu^+^, which in nature can be carried by a number of electron donors, such as small mol­ecules or proteins found in their natural environment (Kracher *et al.*, 2016 ▸; Várnai *et al.*, 2018 ▸; Brenelli *et al.*, 2018 ▸; Frommhagen *et al.*, 2017 ▸; Wang *et al.*, 2020 ▸). *In vitro* reductants such as ascorbic acid, gallic acid or cysteine are commonly used (Wang *et al.*, 2020 ▸). As well as being essential for further reactivity, there are several reports indicating increased polysaccharide affinity on reduction of the active-site metal, suggesting that polysaccharide binding precedes further steps in the mechanism (Kracher *et al.*, 2018 ▸; Hangasky & Marletta, 2018 ▸; Filandr *et al.*, 2020 ▸; Brander, Tokin *et al.*, 2021 ▸). Both O_2_ and H_2_O_2_ have been suggested as natural co-substrates (Bissaro *et al.*, 2017 ▸; Hangasky *et al.*, 2018 ▸) and the LPMO mechanism is being assessed continuously for the involvement of either/both (Bissaro *et al.*, 2020 ▸; Hedegård & Ryde, 2017 ▸; Courtade *et al.*, 2020 ▸; McEvoy *et al.*, 2021 ▸; Brander *et al.*, 2020 ▸). The dependence on H_2_O_2_ for saccharide cleavage has been established recently specifically for one of the model LPMOs investigated here (Brander, Tokin *et al.*, 2021 ▸), and is generally gaining increasing support. The H_2_O_2_-driven mechanism has the advantage that the LPMO active-site metal only needs to be reduced once, while in the O_2_-driven mechanism, it needs to be reduced at every cycle (Bissaro *et al.*, 2017 ▸). However, too high levels of H_2_O_2_ cause oxidative damage and destruction of the enzymes, com­plicating its use as co-substrate (Müller *et al.*, 2018 ▸), and H_2_O_2_ will react with other chemical species, leading to unwanted deca­rboxylating reactions in com­plex substrates, such as lignocellulose, under industrially relevant conditions (Peciulyte *et al.*, 2018 ▸).

For LPMOs, X-rays may possibly circumvent the need for additional reducing agents in structural studies of the reaction (Fig. 1 ▸). Previously, photoreduction of LPMOs has been studied in detail for members of AA10 and AA13 (Gudmundsson *et al.*, 2014 ▸; Muderspach *et al.*, 2019 ▸), and somewhat more qualitatively for AA9 (Frandsen *et al.*, 2016 ▸ ▸).

Here we further investigated the gradual photoreduction of the LPMO active-site Cu^2+^ in a systematic way and monitored the structural changes in two AA9 LPMOs which have been studied extensively. We studied the primarily C4-oxidizing AA9 LPMO from the fungus *Lentinus similis* (*Ls*AA9_A), which is active on soluble cello-oligosaccharides and polysaccharides. We exposed crystals of ligand-free and cello-oligosaccharide-binding *Ls*AA9_A to different radiation doses. To investigate the effect of post-translational modification and/or crystallization conditions on the metal-centre X-ray-induced perturbation, we carried out a similar study with *Ls*AA9_A produced in *E. coli* [*Ls*AA9_A(*Ec*)], thus devoid of glycosyl­ation and His methyl­ation. We determined a total of six structures *per* substrate-free enzyme and six structures *per* enzyme solved with bound cello-oligosaccharide. Additionally, we have solved six structures of the C1/C4-oxidizing AA9 from *Thermoascus auranti­acus* (*Ta*AA9_A, where oligosaccharides bound in the structure have so far not been obtained) to identify similarities between AA9s as the central Cu ion is reduced. We report how inter­atomic distances and other geometric parameters within the active site change as a function of the X-ray dose, and as Cu transitions from Cu^2+^ to Cu^+^. We have correlated our photoreduced structures to the chemically reduced structure of *Ls*AA9_A and two structures solved from data collections at room tem­per­ature. Furthermore, we have monitored photoreduction in a Serial Synchrotron Crystallography (SSX) experiment of *Ls*AA9_A. The results of the study are relevant both for the general study of metalloproteins by X-ray crystallography and the specific understanding of the LPMO mechanism.

## 2. Methods

### 2.1. Crystallization

Crystallization *via* sitting-drop vapour diffusion was performed in MRC two-drop plates set up by an ORYX-8 robot (Douglas Instruments), unless otherwise stated. Cello-oligosaccharides used for substrate-bound structures were purchased from Megazyme.

The fungal enzyme *Ls*AA9_A was expressed in *A. oryzae* [*Ls*AA9_A(f)], purified and deglycosyl­ated with endoH as described previously (Frandsen *et al.*, 2016 ▸; Simmons *et al.*, 2017 ▸; Tandrup *et al.*, 2020 ▸). The protein sample was pre-incubated with Cu^2+^ acetate in equimolar amounts for 1 h at 4°C prior to setup. Crystals of *Ls*AA9_A(f) were grown *via* sitting-drop vapour diffusion. Crystallization and optimization of the crystals has been described previously (Frandsen *et al.*, 2016 ▸). The two crystals used for determining 12 of the structures presented here were grown with a reservoir consisting of 3.3 *M* NaCl and 0.1 *M* citric acid at pH 3.5. Drop and reservoir volumes were 0.5 and 100 µl, respectively, with a drop com­position of 0.3 µl protein (19 mg ml^−1^ in 20 m*M* sodium acetate pH 5.5), 0.1 µl reservoir and 0.1 µl milli-Q water. Crystals appeared within 2 d. Prior to harvesting, the crystals were moved to a drop of 3.0 *M* NaCl and 0.1 *M* citric acid at pH 5.5 for 10 min to ensure an ordered active site (Frandsen *et al.*, 2017 ▸). Subsequent soaking with oligosaccharide ligand was done for one of the crystals by moving it to a drop containing 3.0 *M* NaCl, 0.1 *M* citric acid pH 5.5, as well as 1.0 *M* of Cell_4_ for 10 min. Crystals were flash frozen in liquid nitro­gen without any added cryoprotectant.

The *Ls*AA9_A(f) crystal used for ascorbic acid soak was grown in a VDX plate (Hampton Research) by hanging-drop vapour diffusion. The crystal was grown in a 2 µl drop of 1.5 µl 3.5 *M* NaCl, 0.1 *M* citric acid pH 4.0 and 0.5 µl enzyme (23 mg ml^−1^ in 20 m*M* Na acetate pH 5.5), with a reservoir of 1 ml. After growing a crystal of approximately 200 µm in each dimension, it was transferred to a drop consisting of 3.5 *M* NaCl, 0.1 *M* citric acid pH 5.5, 0.01 *M* ascorbic acid pH 5.5 for 20 min before flash freezing.

For room-tem­per­ature data, an *Ls*AA9_A(f) crystal was grown using 15 µl 20.8 mg ml^−1^ enzyme (Cu-loaded using Cu acetate in equimolar amounts) in 5 µl 3.0 *M* NaCl, 0.1 *M* citric acid pH 3.5 in a VDX plate (Hampton Research). The crystal grew to approximately 200 µm in each dimension within a few weeks and was transferred to a solution of 3.0 *M* NaCl, 0.1 *M* citric acid pH 5.5 for 10 min before being mounted on a cryo-loop (MiTeGen). A MicroRT capillary (MiTeGen) was pre­pared to contain 20 µl of 3.0 *M* NaCl, 0.1 *M* citric acid pH 5.5. The cryo-loop-mounted crystal was inserted into the MicroRT capillary to prevent the crystal from drying during data collection.

A room-tem­per­ature data set was collected at the BioMAX beamline of the MAX IV Laboratory from a crystal grown in 0.1 µl 3.0 *M* NaCl, 0.1 *M* citric acid pH 3.5 and 0.3 µl 19 mg ml^−1^ *Ls*AA9_A(f), also Cu-loaded. The crystal was equilibrated for pH, as above. The crystal was mounted in a 1.0 mm diameter glass capillary (WJM glass), plugged with 3.0 *M* NaCl, 0.1 *M* citric acid pH 5.5 in both ends and sealed with beeswax.

*Ls*AA9_A(*Ec*) was produced in *E. coli* using the LyGo platform with the pLyGo-*Ec*-6 expression vector (Hernández-Rollán *et al.*, 2021 ▸). The sample was purified using Q-Sepharose anion-exchange chromatography with a column equilibrated with 25 m*M* Bis-Tris pH 5.87. The sample was eluted using the same buffer over a 0–500 m*M* NaCl gradient. The protein, in 25 m*M* Bis-Tris pH 5.87 and 175 m*M* NaCl, was initially screened for crystallization conditions at a concentration of 1.83 mg ml^−1^ in 0.3 µl drops (3:1 and 1:1 protein-to-reservoir ratio) with the commercially available Index (Hampton Research), PACT (Qiagen) and JCSG+ (Mol­ecular Dimensions) crystallization screens. At least 30 min before crystallization setup, equimolar quantities of acetate were added to the enzyme sample. Crystals were formed at room tem­per­ature in the A2 conditions of the Index screen [0.1 *M* sodium acetate trihydrate pH 4.5, 2.0 *M* (NH_4_)_2_SO_4_], which was further optimized to 0.1 *M* sodium acetate pH 4.5 and 1.8 *M* (NH_4_)_2_SO_4_. Crystals grown under this condition were flash frozen without added cryoprotectant.

An initial oligosaccharide-binding structure of *Ls*AA9_A(*Ec*)-Cell_3_ was obtained from a crystal soaked for 30 min in a 3 µl drop suspended over a reservoir with 100 µl 2 *M* (NH_4_)_2_SO_4_ and 0.1 *M* sodium acetate in a VDX plate. The drop consisted of 16% glycerol, 0.3 *M* Cell_3_ and *Ls*AA9_A(*Ec*) protein stock to facilitate a final concentration of 0.3 mg ml^−1^. The soaked crystals were mounted in fibre loops and flash frozen.

Crystals of *Ls*AA9_A(*Ec*) soaked in cello­tetra­ose (Cell_4_) were grown by the batch crystallization method in 1.5 ml micro­fuge tubes (Fisher Scientific) using a 1.8 mg ml^−1^ protein concentration. The crystallization condition was 2.3 *M* (NH_4_)_2_SO_4_, 0.1 *M* sodium acetate pH 4.2. A 200 µl batch was set up at 4°C with the protein and the crystallization condition in a 1:3 ratio. The crystals appeared within 4 d. For soaking, 0.5 µl of the crystal slurry was added to a drop containing 0.05 *M* Cell_4_ in the crystallization condition with 18% glycerol and incubated from 5 to 30 min. The crystals were then flash frozen in liquid nitro­gen.

The crystals of *Ls*AA9_A(*Ec*) used for photoreduction were grown in microbatch crystallization plates. Crystals not soaked in substrate were grown in 4 µl drops com­posed of 3 µl 2.0 *M* (NH_4_)_2_SO_4_, 0.1 *M* sodium acetate pH 4.5 and 1 µl *Ls*AA9_A(*Ec*) (1.8 mg ml^−1^ in 25 m*M* Bis-Tris pH 5.8 175 m*M* NaCl). Crystals soaked in Cell_3_ were grown in 3 µl drops com­posed of 2 µl 2.0 *M* (NH_4_)_2_SO_4_, 0.1 *M* sodium acetate pH 4.5 and 1 µl *Ls*AA9_A(*Ec*) (1.8 mg ml^−1^ in 25 m*M* Bis-Tris pH 5.8, 175 m*M* NaCl). The drops were covered in a layer of paraffin and silicon oils in a paraffin–silicon ratio of 3:2. The drops were set up at 4°C and crystals appeared within 4 d. Crystals used in a soaking experiment were moved into a drop containing 0.5 *M* Cell_3_, 2.0 *M* (NH_4_)_2_SO_4_ and 0.1 *M* sodium acetate pH 4.5. Prior to flash freezing in liquid nitro­gen, the crystals were cryoprotected in 20%(*w*/*v*) glycerol, 2.0 *M* (NH_4_)_2_SO_4_, 0.1 *M* sodium acetate pH 4.5.

*Ta*AA9_A was produced as described in Harris *et al.* (2010 ▸) and purified using Q-Sepharose anion-exchange chromatography with a column equilibrated with 20 m*M* Tris pH 6.0. *Ta*AA9_A was eluted in the same buffer over a 0.79–2.5 m*M* NaCl gradient. The sample was deglycosyl­ated in 20 m*M* MES pH 6.0, 125 m*M* NaCl by incubation with ∼0.05 units per mg *Ta*AA9_A of endoH (Roche Diagnostics, 11643053001), and then buffer exchanged to 20 m*M* Na acetate pH 5.5. Prior to crystallization the sample was incubated for 1 h with equimolar amounts of Cu acetate. Crystals of *Ta*AA9_A were grown in 0.4 µl drops com­posed of 0.3 µl *Ta*AA9_A (15.9 mg ml^−1^ in 20 m*M* sodium acetate pH 5.5) and 0.1 µl 0.02 *M* MgCl_2_, 0.1 *M* HEPES pH 7.5, 22%(*w*/*v*) polyacrylic acid 5100 sodium salt.

### 2.2. Single-crystal X-ray data collection, structure determination and refinement

Data for the initial structure determination of *Ls*AA9_A(*Ec*) were collected at 100 K at the BioMAX beamline at MAX IV (Lund, Sweden) (Ursby *et al.*, 2020 ▸). The images were col­lected for 360° with an oscillation of 0.1° and a 100 ms exposure time for each image using a wavelength of 1.1 Å. The BioMAX beamline was also used to collect 360° data with an oscillation of 0.1° and an 11 ms exposure time for each image for *Ls*AA9_A(*Ec*)-Cell_4_ crystals. The autoprocessed (autoPROC pipeline) reflection file was used for structure determination. The highest resolution cut-off of diffraction data was generally chosen based on a CC_1/2_ in the outer shell of above 50%.

For *L*sAA9_A(f), a data set was collected at room temper­ature using an in-house diffractometer at the Oak Ridge National Laboratory. The set-up consisted of a Rigaku HighFlux HomeLab instrument with a MicroMax-007 HF X-ray generator and an EIGER R 4M detector. Data were collected for 98° with 0.25° oscillation and a 10 s exposure per image. The data were processed using *CrysAlis PRO* (Rigaku) and scaled using *AIMLESS*/*POINTLESS* (Evans & Murshudov, 2013 ▸; Evans, 2006 ▸). Room-tem­per­ature data were also collected for *Ls*AA9_A(f) at the BioMAX beamline of the MAX IV synchrotron. The glass capillary mounted sample was illuminated with X-rays for 360° with an oscillation of 0.1° and an exposure per frame of 0.01 s. The data were processed using *XDS*/*XSCALE*.

Data for the photoreduction study were collected at the P11 beamline of PETRA-III at DESY, Hamburg (Burkhardt *et al.*, 2016 ▸). For each of the data sets, images were collected for 360° with an oscillation of 0.1° and a 100 ms exposure time for each image. Three data sets were collected on *Ls*AA9_A(f) and *Ls*AA9_A(f)-Cell_4_ with the beam transmission set to 1, 10 and 100%. This corresponded to estimated photon fluxes of 10^10^, 10^11^ and 10^12^ photons/s, respectively. Each data collection was performed without changing the crystal orientation between collections. Subsets of the three data sets (two for each) were processed with the fewest number of images possible for a com­pleteness of ∼90%. The number of images for each data set was limited in an effort to trap the structures with a specific oxidation state of the Cu, thus being the basis for six structures per crystal, each at a different dose. The structures corresponding to the *Ls*AA9_A(f) data sets were solved according to Table 1 ▸. The collection strategy was identical between the data sets collected from substrate-free crystals, and crystals soaked in Cell_4_. For both the *Ls*AA9_A(f) structures with and without substrate, anomalous signal could be used to determine the absence/presence of Cl^−^ at the active site.

Data-collection parameters for the photoreduction of crystals of *Ls*AA9_A(*Ec*) and *Ta*AA9_A were chosen to approximately match the relative doses received by the *Ls*AA9_A(f) crystals described above. Using *RADDOSE3D* (Zeldin *et al.*, 2013 ▸), the average diffraction weighted dose was calculated prior to X-ray exposure to find the optimal flux required to achieve similarly reduced active-site Cu in the resulting structures and calculated again after the experiment for a final dose.

Data for the photoreduction study were also processed and scaled using *XDS*/*XSCALE* (Kabsch, 2010 ▸). The lowest dose data sets were used as references during the processing of higher dose data sets to ensure consistent indexing. The highest resolution cut-off was generally chosen based on a CC_1/2_ in the outer shell of above 40%. The consequence of following the set scheme in Table 1 ▸ was that in some cases a com­pleteness of less than 100% and a data redundancy <2 were obtained, but this was considered a reasonable trade-off for consistency. Phases for *Ls*AA9_A(f) were obtained from the previously published high-resolution *Ls*AA9_A(f) structure (PDB entry 5ach) (Frandsen *et al.*, 2016 ▸). For *Ls*AA9_A(*Ec*), the space group was different than for *Ls*AA9_A(f), and the phases were obtained using *MOLREP* (Vagin & Teplyakov, 1997 ▸) and PDB entry 5ach as the search model. Phases for *Ls*AA9_A(*Ec*)-Cell_4_ and Cell_3_ were obtained from the isomorphous oligosaccharide-free structures. For *Ta*AA9_A, the phases were obtained from the previously published high-resolution *Ta*AA9_A structure with a single mol­ecule in the asymmetric unit (PDB entry 3zud) (Quinlan *et al.*, 2011 ▸).

For all structures, rigid-body and restrained refinement were performed in *REFMAC5* (Murshudov *et al.*, 1997 ▸) of the *CCP*4 suite (Winn *et al.*, 2011 ▸), alternated with manual rebuilding and validation performed in *Coot* (Emsley & Cowtan, 2004 ▸). Further validation was performed using *BAVERAGE* and *PROCHECK* (Vaguine *et al.*, 1999 ▸) of the *CCP*4 suite (Winn *et al.*, 2011 ▸). For data collection and refinement statistics, see Tables S1–S6 in the supporting information. Figures of the resulting models were prepared in *PyMOL* (*The PyMOL Mol­ecular Graphics System*; Version 2.0.4; Schrödinger, LLC). The active-site distances and angles of the final models were measured in *Coot* and using the Python library Biopython (Cock *et al.*, 2009 ▸). Animations of active-site changes during photoreduction (see Movies S1–S5 in the supporting information) were made in *PyMOL* using the *morph* feature with no trajectory refinement.

### 2.3. Serial synchrotron crystallography

The *Ls*AA9_A(f)-SSX data set was collected at the ID29 beamline (de Sanctis *et al.*, 2012 ▸) of the European Synchrotron Radiation Facility (ESRF). The data were collected using the *Mesh & Collect* routine (Zander *et al.*, 2015 ▸), in a similar manner to that described recently for *Ao*AA13 (Muderspach *et al.*, 2019 ▸). For each crystal identified by the routine, data were collected over a total rotation of 10°, though only 2.5° were used for processing, with an oscillation of 0.1° per image at a wavelength of 0.98 Å. 13 data sets were processed individually with identical parameters using *XDS* (Kabsch, 2010 ▸). The hierarchical cluster analysis software *ccCluster* (Santoni *et al.*, 2017 ▸) was used to determine which data sets could be merged and scaled based on their correlation coefficient distances. The resulting data set from this merge was used to solve the *Ls*AA9_A(f)-SSX structure. Phasing and refinement were performed as described above.

## 3. Results and discussion

### 3.1. Overall structure overview for this study

This study includes five LPMO and substrate combinations with similar photoreduction protocols at cryogenic tem­per­atures: *Ls*AA9_A produced in two recombinant systems giving different post-translational modifications and crystallized under different conditions in different space groups, in the presence/absence of oligosaccharide ligands, and *Ta*AA9_A (without the oligosaccharide ligand). Table 2 ▸ summarizes the post-translational modifications, crystallization solution com­positions, crystal form characteristics and exogenous ligands for the five combinations investigated. *Ls*AA9_A and *Ta*AA9_A display the immunoglobin G-like β-sandwich fold observed for all LPMOs structurally characterized so far [see Figs. 1 ▸(*b*) and 1(*c*)]. The catalytic Cu ion is coordinated by the His-brace, fitting an elongated octa­hedral geometry, and lies in the middle of a relatively flat surface (Frandsen & Lo Leggio, 2016 ▸; Vu & Ngo, 2018 ▸; Tandrup *et al.*, 2018 ▸), where the polysaccharide substrate has been demonstrated experimentally to bind in several AA9 LPMOs (Frandsen *et al.*, 2016 ▸; Tandrup *et al.*, 2020 ▸; Courtade *et al.*, 2016 ▸). In both proteins, a tyrosine –OH group functions as one of the axial ligands to the Cu. In oligosaccharide-free *Ls*AA9_A(f), the copper coordination sphere includes two water ligands in equatorial (eq) and axial (ax) positions (see Fig. 1 ▸). The chemical nature of the exogeneous equatorial and axial ligands changes depending on the crystallization conditions and the exogenous axial ligand is displaced upon oligosaccharide binding.

The bacterially expressed *Ls*AA9_A(*Ec*) has recently been shown to be fully active (Brander, Tokin *et al.*, 2021 ▸) and was also used in a high-resolution study to determine the proton­ation stages in the second coordination sphere of Cu (Banerjee *et al.*, 2022 ▸). The structure of *Ls*AA9_A(*Ec*) is very similar to the previously published fungally expressed *Ls*AA9_A(f) structures (all-atom r.m.s. deviation for resting-state substrate-free structures: overall = 0.56 Å and His-brace = 0.10 Å), except for post-translational modifications and differences in the exogenous ligands to the Cu due to different crystallization conditions (Table 2 ▸ and Fig. S1).

The crystal packing around the substrate-binding site differs between the *Ls*AA9_A(*Ec*) and *Ls*AA9_A(f) crystal forms. *Ls*AA9_A(f) has an unrestricted binding site, so that *Ls*AA9_A(f) crystals soaked in Cell_4_ in this study bind from subsite −2 to +2, as reported previously (Tandrup *et al.*, 2020 ▸). In contrast, the −2 binding subsite of *Ls*AA9_A(*Ec*) is blocked by crystal contacts. This prevents binding in the same mode observed previously for *Ls*AA9_A(f) for both Cell_3_ and Cell_4_ (Frandsen *et al.*, 2016 ▸; Tandrup *et al.*, 2020 ▸). In the structure of *Ls*AA9_A(*Ec*)-Cell_4_, the substrate is modelled from subsite −1 to +3, with the glucose monomer in the +3 subsite being partially disordered (see Figs. S2 and S3). Thus, we have chosen *Ls*AA9_A(*Ec*)-Cell_3_ as a model for the photoreduction study. In this structure, Cell_3_ is bound at subsites −1 to +2. In line with previous structural studies (Frandsen *et al.*, 2016 ▸), the binding of oligosaccharides expels the axial water mol­ecule, thus only the equatorial exogenous ligand remains. A Cl^−^ ion occupies this position where O_2_ or H_2_O_2_ are expected to bind during the reaction. For *Ls*AA9_A(f), this is likely due to the high NaCl concentration in the crystallization conditions (upwards of 3.0 *M*), while for *Ls*AA9_A(*Ec*), the NaCl concentration is much lower. Anomalous signal, routinely used to detect heavy atoms in crystals, could only determine the presence of Cl^−^ unambiguously in *Ls*AA9_A(f)-Cell_4_ structures (not shown), but Cl^−^ still fits the observed electron density more accurately for *Ls*AA9_A(*Ec*)-Cell_3_, com­pared to water and SO_4_^2−^.

The structure of *Ta*AA9_A presented here is similar to those reported previously (PDB entries 3zud and 2yet) (Quinlan *et al.*, 2011 ▸), except that the active-site copper is well defined and free of the disorder observed previously. *Ta*AA9_A has been used previously in describing the LPMO active site in theoretical studies (Hedegård & Ryde, 2018 ▸; Kjaergaard *et al.*, 2014 ▸; Kim *et al.*, 2014 ▸) and the structures here could therefore present an improvement for further calculations. Crystal contacts most likely prevent binding at negative subsites, assuming the oligosaccharide binds in a similar manner to previous oligosaccharide-binding structures with *Ls*AA9_A and an AA9 from *Collariella virescens* (*Cv*AA9_A) (Frandsen *et al.*, 2016 ▸; Simmons *et al.*, 2017 ▸; Tandrup *et al.*, 2020 ▸), and it has not been possible so far to obtain an oligosaccharide bound in the structure of this protein, which also has no reported activity on oligosaccharides.

Crystals of fungal and bacterially expressed *Ls*AA9_A (unsoaked and soaked in Cell_4_ and Cell_3_ oligosaccharide, respectively) and crystals of *Ta*AA9_A were exposed to X-rays for three consecutive data collections, with an increasing flux of photons, and from these, images were selected to form data sets for structure determination at individual X-ray doses. The images in each data set were limited to the minimal amount necessary to solve the structures while still obtaining a com­plete and statistically reas­onable data set (see *Methods* section for details of the data reduction and Tables S1–S6 for statistics). We solved two structures from each of the three collections for all the crystals, giving snapshots at six X-ray doses for each. Two additional low-dose structures for the study come from the data for *Ls*AA9_A(f) at room tem­per­ature (RT) from an in-house diffractometer and synchrotron to resolution limits of 1.8 and 1.9 Å, respectively (see Fig. S4). As a reference for the Cu^+^ state, we used a chemically reduced structure obtained from *Ls*AA9_A(f) crystals soaked in 10 m*M* ascorbic acid.

These reference structures were used to establish which parameters should be monitored to follow the transition from Cu^2+^ to Cu^+^. Previously, a tendency towards a slight increase in the Cu—His1-N distances and slight decreases in the Cu—His1 N^δ1^ and Cu—His N^ɛ2^ distances have been reported on photoreduction (Gudmundsson *et al.*, 2014 ▸). However, as can be seen in Table 3 ▸, the differences are very small between our Cu^2+^ and Cu^+^ reference structures. The transition from Cu^2+^ to Cu^+^ can instead be followed more clearly by measuring the inter­atomic distances between Cu and its ligands (see Table 3 ▸), including the equatorial and axial water mol­ecules (see Fig. 1 ▸). In terms of distances, we here define the Cu state based on distances <2.2, 2.2–2.9 and >2.9 Å between Cu and the equatorial ligand as Cu^2+^, a mix and Cu^+^, respectively. For the axial Cu ligand, the distances are defined here as <2.7, 2.7–3.2 and >3.2 Å, respectively. While due to Jahn–Teller distortion (Jahn & Teller, 1937 ▸) even longer distances have been observed, at distances longer than these we can probably be confident that there is no longer a coordination bond. Compared to earlier studies on LPMOs, the lowest-dose Cu–water ligand distances found here are slightly longer than for other type II Cu sites investigated using X-ray doses in a similar range (Frankaer *et al.*, 2014 ▸). For reference, in aqueous six-coordinated com­plexes of Cu^2+^, equatorial water mol­ecules are at a distance of around 2.0 Å from the ion and axial water mol­ecules are up to 0.3 Å longer (de Almeida *et al.*, 2009 ▸). These distances have been used previously to examine the active-site geometry of LPMOs (Vu & Ngo, 2018 ▸), and the definitions of distance magnitudes are based on previous LPMO studies and small-mol­ecule Cu com­plexes (Gudmundsson *et al.*, 2014 ▸; Vu & Ngo, 2018 ▸; Persson, 2010 ▸). Based on these cut-offs, our reference low-dose *Ls*AA9_A(f) structure is very close to a full Cu^2+^ state. For the chemically reduced crystals, structures at two X-ray doses were obtained (a low dose of 2.08 × 10^3^ Gy and a high dose of 1.70 × 10^7^ Gy) (see Fig. S5). Consistent with a fully Cu^+^ state, the distances to the equatorial and axial water mol­ecules are large even at low dose (4.0 and 3.7 Å) and do not increase with dose (4.0 and 3.5 Å, respectively) (see Table 3 ▸ for all measured active-site distances).

Upon reduction of the metal from Cu^2+^ to Cu^+^, the geometry is expected to change from a trigonal bipyramid, distorted square pyramid, seesaw or elongated octa­hedral geometry (the latter applicable to the AA9 LPMOs studied here) towards a T-shaped geometry, with increased deviations from 90° in the defined angles θ_1_ and θ_2_, a decrease in θ_3_ and an increase in θ_T_ [see Fig. 1 ▸(*d*)] (Vu & Ngo, 2018 ▸). The θ_1_, θ_2_, θ_3_ and θ_T_ values in the chosen reference structures are consistent with the previous findings for the geometrical differences between Cu^2+^ and Cu^+^ geometries, and a steady increase in θ_2_ and a decrease in θ_3_ are the clearest trends which are common to the three types of crystals investigated. We have further observed a decrease in θ_H–H_ and θ_H1_, with an increase in θ_HN_ in the fully chemically reduced structures com­pared to the reference Cu^2+^ structure (see Fig. S6). Most angles do not change upon continued exposure of the chemically reduced *Ls*AA9_A(f), except θ_H1_ and θ_H–H_, which decrease further (see Table S7). Thus, all these angles have also been monitored through the photoreduction studies as useful indicators of the Cu ion state (Tables S7 and S8).

### 3.2. Photoreduction of substrate-free AA9s

**Figure 2:**
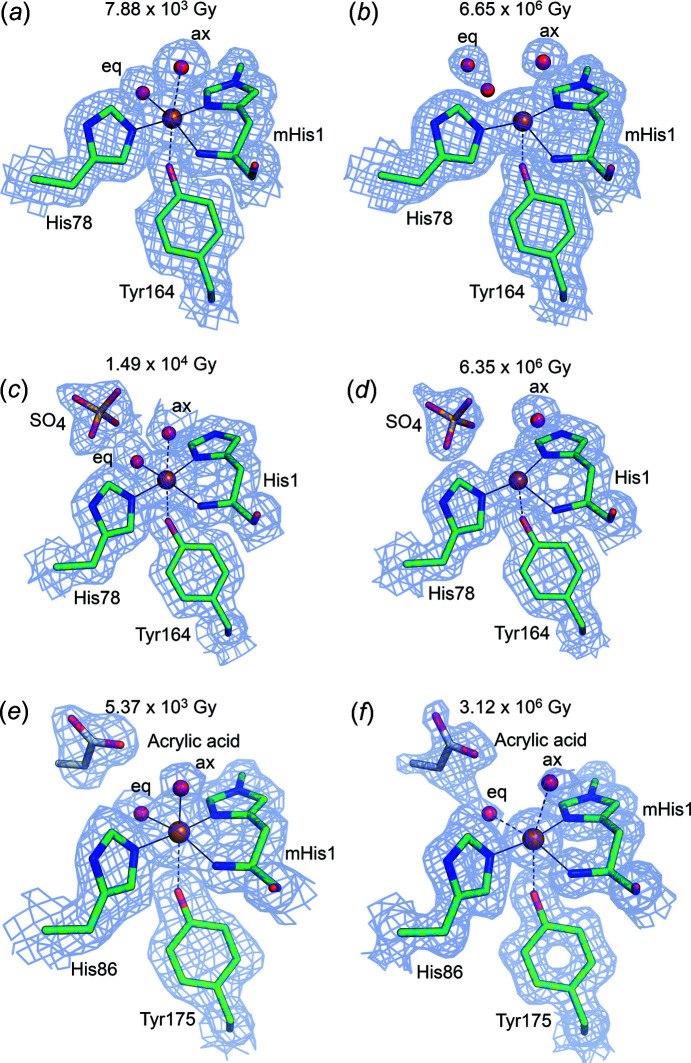
LPMO Cu sites in *Ls*AA9_A(f), *Ls*AA9_A(*Ec*) and *Ta*AA9_A at the lowest and highest X-ray doses. *Ls*AA9_A(f) at (*a*) low and (*b*) high dose. *Ls*AA9_A (*Ec*) at (*c*) low and (*d*) high dose. *Ta*AA9_A at (*e*) low and (*f*) high dose. The distances between Cu (orange spheres) and the equatorial/axial water mol­ecules (red spheres) increase with increasing X-ray dose in the transition from Cu^2+^ to Cu^+^. In some cases, the water mol­ecules have been modelled in a double conformation (*b*) or left unmodelled due to a lack of electron density (*d*). Distances below 2.2/2.7 Å are shown as full lines (coordination distance), below 2.9/3.2 Å as dashed lines (close to coordination distance) and above 2.9/3.2 Å with no line (not coordinating) for the equatorial/axial ligands, respectively. 2*F*_o_–*F*_c_ electron density is shown at a 1.0 contour level as blue mesh.

The lowest dose *Ls*AA9_A(f) cryotem­per­ature structure was solved from a data set totaling a dose of 7.88 × 10^3^ Gy [Figs. 1 ▸ and 2 ▸(*a*)]. Detailed transitions (all X-ray doses) can be seen in Figs. S7 and S8, and Movies S1 and S2, while different geometrical parameters under photoreduction are monitored in Figs. S6 and S9. Efforts were made to expose other crystals to com­parable doses. For *Ls*AA9_A(*Ec*), the lowest dose was 1.49 × 10^4^ Gy [Fig. 2 ▸(*c*)]. In both cases, the active-site distances and angles agree well with a Cu^2+^ oxidation state of type II Cu (Vu & Ngo, 2018 ▸; Solomon *et al.*, 2014 ▸; Frandsen *et al.*, 2016 ▸) [see Figs. 2 ▸(*a*) and 2(*c*)], although θ_3_ and θ_T_ may indicate that photoreduction has started for *Ls*AA9_A(*Ec*), which is collected at a slightly higher dose. In all the low-dose structures, Cu has been modelled in full occupancy. The distance between Cu and the equatorial water is 0.2 Å shorter in *Ls*AA9_A(*Ec*) and is probably affected by a nearby sulfate ion at a hydrogen-bond distance of 2.5 Å. The key measured geometrical parameters are presented in Table 3 ▸, with an additional analysis in Tables S7 and S8. In the lowest-dose substrate-free structures of fungal/bacterial *Ls*AA9_A, the distance to the equatorial water ligand is in the range 2.2–2.3 Å, while that to the axial ligand is in the range 2.6–2.7 Å. For com­parison, QM/MM models have shown distances of 2.16/2.25, 2.08/2.53 and 2.21/2.34 Å for the equatorial/axial water mol­ecule, depending on the parameters of the calculation (Theibich *et al.*, 2021 ▸), which could indicate that even at the lowest X-ray dose possible, a small amount of photoreduction has occurred.

**Figure 3:**
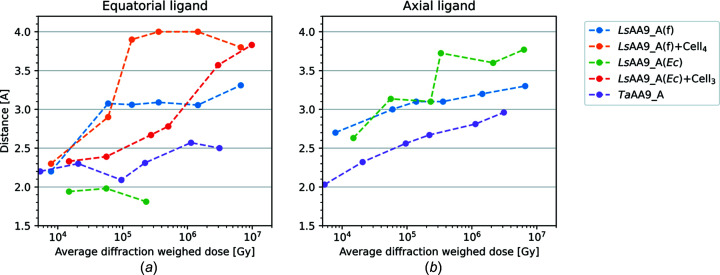
Measured distances as a function of the average diffraction weighted radiation dose. Distances are measured from the Cu atom to either the equatorial ligand or the axial water mol­ecule. In substrate-binding structures, no measurement is taken for the axial water, as it has been displaced by substrate. For *Ls*AA9_A(*Ec*), the density for the equatorial water disappears com­pletely after the third dose structure. All distances are listed in Table S7.

As the X-ray dose increases, so do the distances to the exogenous water ligands, which gradually become disordered and/or disappear, as expected (see Fig. 3 ▸). For all inter­mediate doses of *Ls*AA9_A(f), the equatorial water mol­ecule has been modelled in a double conformation, each with 50% occupancy. For *Ls*AA9_A(*Ec*), the equatorial water mol­ecule becomes disordered at an X-ray dose above 3.33 × 10^5^ Gy, where the mol­ecule can no longer be modelled confidently. At inter­mediate doses of *Ls*AA9_A(*Ec*), the axial water is modelled in a double conformation, each with 50% occupancy, or in an alternative conformation with sulfate (see Fig. S8), likely hindering the migration of the water.

Both *Ls*AA9_A(f) and *Ls*AA9_A(*Ec*) structures with the highest X-ray doses (6.65 × 10^6^ and 6.35 × 10^6^ Gy, respectively) exhibit exogenous ligand distances and other parameters agreeing with a predominant Cu^+^ state, as reported previously (Vu & Ngo, 2018 ▸; Gudmundsson *et al.*, 2014 ▸; Frandsen *et al.*, 2016 ▸) and in agreement with our chemically reduced *Ls*AA9_A(f) structures. In *Ls*AA9_A(f), both the axial and the equatorial water mol­ecules have increased distances from Cu com­pared to the lowest dose (0.6 Å more for the axial and 1.2 Å more for the equatorial) and >3.3 Å from Cu. Thus, the Cu atom has effectively lost its two ligands when reduced from Cu^2+^ to Cu^+^ [see Table 3 ▸ and Figs. 2 ▸(*b*) and S7]. In the higher-dose structures, the equatorial water mol­ecule has migrated away from the Cu ion and is coordinated by Gln162 and/or His147 of the secondary coordination sphere instead (Fig. S10). In *Ls*AA9_A(*Ec*), there is no residual density for the equatorial water at the highest dose, while the axial water is 0.5 Å further away than in the lowest dose. Compared to the chemically reduced crystals, the measured Cu-site distances are shorter even in the highest dose *Ls*AA9_A(f) structure. This might imply that the *Ls*AA9_A(f) structure is not fully reduced and that it may indeed be difficult to fully photoreduce an LPMO. Alternatively, photoreduction at 100 K may impede full migration of bound water.

**Figure 4:**
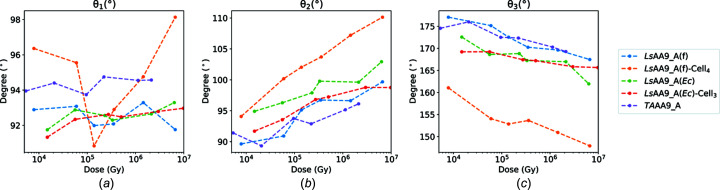
Measured active-site angles θ_1_, θ_2_ and θ_3_ as a function of the average diffraction weighted radiation dose. A consistent observation for all proteins in the photoreduction study is an increase in θ_2_ and a decrease in θ_3_, while θ_1_ is less consistent. All angles are listed in Table S7.

Looking at the θ angles in the two substrate-free *Ls*AA9_A variants (Figs. 4 ▸ and S6), Cu reduction is associated with an increase in θ_2_, θ_T_ and θ_HN_ (>8, >3 and >3°, respectively). Additionally, a decrease in θ_3_, θ_H–H_ and θ_H1_ (>9, >3 and >9°, respectively) can be observed over the transition, although the absolute values between the two enzymes differ somewhat.

In a previous EXAFS study, it was indicated that the Cu^2+^ structure of *Ta*AA9_A could be well represented by four N/O ligands at an average distance from Cu of 1.98 Å (Kjaergaard *et al.*, 2014 ▸), whereby one of the close ligands was lost on photoreduction, leaving the Cu atom in a T-shaped coordination. The equatorial ligand distances in the lowest-dose *Ta*AA9_A structure presented have an average of 2.1 Å, which is within the agreement level that can be expected given an estimated coordinate error of 0.207 Å for this structure (see Table 3 ▸); however, in the crystal structure, an axial water is additionally very close at a distance from Cu of 2.0 Å and is significantly closer than in the *Ls*AA9_A substrate-free structures above. The distance increases steadily with dose to 3.0 Å [see Figs. 2 ▸(*e*) and 2(*f*), with a more detailed transition in Fig. S11 and Movie S3]. The equatorial water mol­ecule sits at 2.2 Å from the Cu atom in the lowest-dose structure and reaches the longest distance of 2.6 Å at an inter­mediate dose (1.13 × 10^6^ Gy). Similar to *Ls*AA9_A(*Ec*), the structure of *Ta*AA9_A has a small mol­ecule (modelled as acrylic acid) near the equatorial position [see Figs. 2 ▸(*e*) and 2(*f*)], and additionally, a HEPES mol­ecule near the axial position, present from crystallization conditions. It is therefore possible that the presence of these ligands affects the observed distances. However, the axial distance in the EXAFS study may also be affected by some photoreduction even when precautions were taken. *Ta*AA9_A may also be slightly less sensitive to photoreduction than *Ls*AA9_A, as the changes in θ values are slightly smaller for the *Ta*AA9_A transition (Figs. 4 ▸ and S6).

For all three substrate-free enzymes used in the photoreduction study, we have obtained a reasonably consistent picture of transition from Cu^2+^ to Cu^+^ in diverse AA9 LPMOs under different crystallization conditions. We observe a loss in coordination between Cu and water ligands at higher doses, changing the coordination from an elongated hexa­coordinated geometry to a T-shaped geometry with Cu coordinated solely by protein ligands, as discussed previously (Gudmundsson *et al.*, 2014 ▸; Vu & Ngo, 2018 ▸; Kjaergaard *et al.*, 2014 ▸). Previous studies of *Efa*CBM33/*Ef*AA10, using doses in a com­parable range to what has been used here (Gudmundsson *et al.*, 2014 ▸), showed that at doses higher than 5.94 × 10^5^ Gy the exogenous water ligands were expelled from the initial trigonal bipyramidal geometry. This points towards the T-shaped geometry being essential when accepting the oxygen co-substrate, despite the initial difference in the water-ligand coordination com­pared to the investigated AA9s. A structure of *Ao*AA13, which has similar Cu geometry to AA9, solved with an X-ray dose of 3.19 × 10^4^ Gy, exhibits a fully reduced Cu site with no visible solvent-facing ligands, and already at the lowest dose (2.57 × 10^3^ Gy) has lost the axial water ligand (Muderspach *et al.*, 2019 ▸), suggesting it is more prone to photoreduction than the analyzed AA9s and AA10.

### 3.3. Serial synchrotron crystallography and room-tem­per­ature structures of *Ls*AA9_A

Serial synchrotron crystallography (SSX) experiments are an effective method for reducing radiation damage com­pared to a conventional MX experiment (de la Mora *et al.*, 2020 ▸; Ebrahim *et al.*, 2019 ▸; Mehrabi *et al.*, 2021 ▸) and could allow better control of photoreduction in metalloproteins. Data collected on 13 crystals were scaled together, resulting in the 2.4 Å *Ls*AA9_A(f)-SSX data set. The dose received by the resulting structure (7.02 × 10^4^ Gy) is com­parable to the oligosaccharide-free *Ls*AA9_A(f) structure with a dose of 5.99 × 10^4^ Gy, and indeed shows a similar geometry, indicating close to full photoreduction, though with a somewhat sharper θ_1_ and wider θ_3_ angle. The θ_T_ angle in the SSX structure is greater than in any of the six *Ls*AA9_A(f) substrate-free structures, or the chemically reduced structures. The axial water mol­ecule was found at 2.9 Å in the *Ls*AA9_A(f)-SSX structure, which agrees with the substrate-free *Ls*AA9_A(f) structure. However, the equatorial water mol­ecule found at 3.9 Å is very indicative of a fully reduced Cu site, despite efforts to limit the dose experienced by each crystal. In part, this could be due to the water in the *Ls*AA9_A(f)-SSX structure having only a single conformation at this position (see Figs. S7 and S12). Perhaps unsurprisingly, SSX does not protect from photoreduction at a similar total dose and it would have been difficult to obtain a lower dose with the applied protocol. The distances observed within the active site are listed in Table 3 ▸, and in Tables S7 and S8.

We collected room-tem­per­ature data for the *Ls*AA9_A(f) crystals, both from an in-house diffractometer and from a syn­chro­tron, at the lowest possible dose (Fig. S4). Here *Ls*AA9_A(f) (RT) from the in-house data has distances of 2.2 and 2.8 Å for the equatorial and axial ligands, respectively, which correlate well with a Cu^2+^ state (Vu & Ngo, 2018 ▸; Gudmundsson *et al.*, 2014 ▸; Frandsen *et al.*, 2016 ▸). Perhaps surprisingly, data collected from a synchrotron source at room tem­per­ature was similarly able to produce a structure containing a Cu^2+^ site [*Ls*AA9_A(f), RT-sync], based on the Cu–water distances and θ_1_–θ_3_ angles (some of the other parameters resemble more a Cu^+^ site). It was in this case possible to achieve a much lower dose than for the corresponding cryogenic tem­per­ature structures (1.91 × 10^3^ Gy).

Due to the rapid decay of the diffraction intensity without cryocooling, a fully photoreduced structure could not be determined using this strategy. Curiously, the *Ls*AA9_A(f) (RT) structure contains a Cl^−^ ion in the axial position, which was confirmed by an anomalous signal (see Fig. S4), which in *Ls*AA9_A(f) (RT-sync) appears to be the more regularly observed water ligand.

### 3.4. Photoreduction of *Ls*AA9_A crystals soaked with oligosaccharides

**Figure 5:**
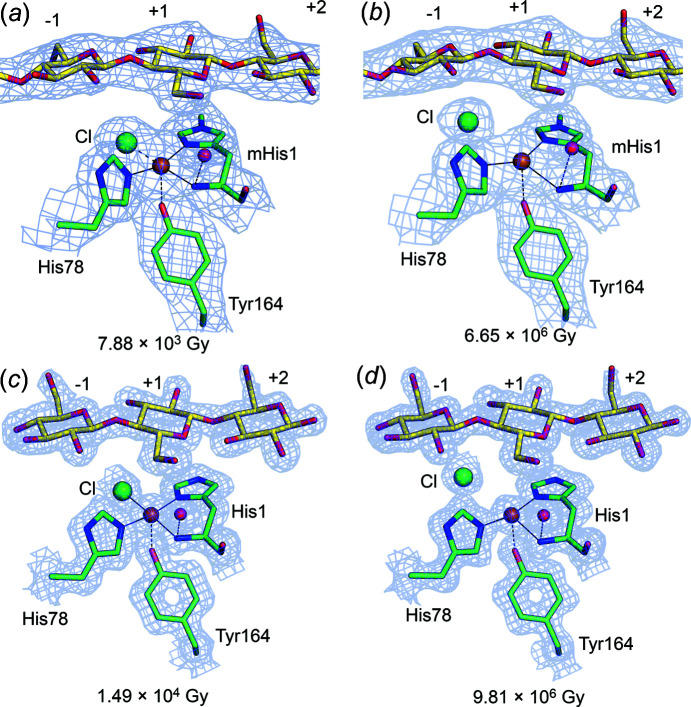
LPMO Cu sites in the oligosaccharide-bound structures of *Ls*AA9_A(f) [(*a*) 7pyd and (*b*) 7pyi] and *Ls*AA9_A(*Ec*) [(*c*) 7pyu and (*d*) 7pz0] at the lowest and highest X-ray doses. For both *Ls*AA9_A(f) and *Ls*AA9_A(*Ec*) at higher X-ray doses, the equatorial Cl^−^ ion (green spheres, present from crystallization conditions) increases its distance relative to the Cu atom. The Tyr164-O to Cu distance is, in all cases, shorter than for the substrate-free structures presented in Fig. 2 ▸. Active-site distances are listed in Table 3 ▸. 2*F*_o_–*F*_c_ electron density is shown at a 1.0 contour level as blue mesh.

Data on *Ls*AA9_A crystals soaked in cello-oligosaccharides were collected using the same protocol as for the oligosaccharide-free crystals. As for the unbound structures, methyl­ation of His1 does not seem to affect photoreduction to any significant extent, nor does it seem to have a structural effect on substrate binding (Frandsen *et al.*, 2016 ▸). For both the *Ls*AA9_A(f) and *Ls*AA9_A(*Ec*) oligosaccharide-bound/unbound structures, the so-called pocket water increases its distance to His1-N with increasing X-ray dose (Figs. 2 ▸, 5 ▸ S7, S9, S10, S13 and S14). Here also the distance from the equatorial ligand to Cu increases with increasing X-ray dose experienced by the crystal (Tables 3 ▸ and S7, and Fig. 5 ▸).

At the lowest doses [Figs. 5 ▸(*a*) and 5(*c*)], the equatorial solvent-facing ligand, modelled as a Cl^−^ ion, is 2.3 Å from the Cu atom, while in the highest-dose structures, the distance has increased to 3.9 and 3.8 Å for *Ls*AA9_A(f)-Cell_4_ and *Ls*AA9_A(*Ec*)-Cell_3_, respectively. For com­parison, the average distance to the equatorial ligand is 2.1 Å between different QM/MM models in a recent theoretical study (Theibich *et al.*, 2021 ▸). In several inter­mediate X-ray doses of *Ls*AA9_A(f)-Cell_4_, the Cl^−^ ion was modelled in a double conformation, with 90% occupancy for the conformation furthest from the Cu atom and 10% occupancy for the conformation closest to the Cu atom, indicating a mixed state between Cu^2+^ and Cu^+^. The distance from the Cl^−^ ion to Cu increases much more rapidly with dose in the *Ls*AA9_A(f)-Cell_4_ structures (3.8 Å at 3.60 × 10^5^ Gy) com­pared to *Ls*AA9_A(*Ec*)-Cell_3_ (3.8 Å at 9.81 × 10^6^ Gy) (Figs. S13 and S14, Table S7, and Movies S4 and S5). It is unclear if these differences are due to the differences between the fungally and bacterially expressed enzymes, or if it is a result of the conditions under which the crystals were grown. At any rate, while in the case of the substrate-free *Ls*AA9_A(*Ec*) structure the equatorial position was partially occupied by sulfate (and partially by a water mol­ecule), this does not seem to be the case for the equatorial ligand at *Ls*AA9_A(*Ec*)-Cell_3_, which is most consistent with fully occupied chloride, although this could not be confirmed with an anomalous signal.

As for the transition from Cu^2+^ to Cu^+^ in the unbound structures, for the oligosaccharide-bound structures there is a clear increase in the θ_2_ angle and a clear decrease in the θ_3_ angle (see Fig. 4 ▸). Furthermore, all the oligosaccharide-bound structures at any dose have values considerably greater than 0 and greater than those of the corresponding unbound structures for the θ_T_ angle, *i.e.* 18.7 ± 3.3° for *Ls*AA9_A(f) and 8.7 ± 0.9° for *Ls*AA9_A(*Ec*) (see Fig. S6). Thus, a significantly greater θ_T_ angle seems to be a characteristic of oligosaccharide-bound *Ls*AA9_A. To further test this hypothesis, we calculated the average value for all the available structures of *Ls*AA9_A with bound saccharide ligands, excluding xylooligosaccharides, as they are questionable as true LPMO substrates (Simmons *et al.*, 2017 ▸). The average was 11.49 ± 2.03° (PDB entries 5aci, 5acf, 5acj, 5n05, 5nkw, 5nlr, 5nls and 6ydg). As θ_T_ also increases with dose in the unbound structures, a better com­parison may be between low-dose structures for all saccharide-free and saccharide-bound *Ls*AA9_A structures to date (Table S9), which also shows a greater θ_T_ value for bound (12.0 ± 3.1°) com­pared to free (2.4 ± 0.9°).

Several reports have suggested a higher affinity of at least some AA9 LPMOs for cellulosic substrates in their Cu^+^ as opposed to their Cu^2+^ form (Kracher *et al.*, 2018 ▸; Bertini *et al.*, 2018 ▸; Courtade *et al.*, 2016 ▸; McEvoy *et al.*, 2021 ▸). This has also been demonstrated recently for *Ls*AA9_A (Brander, Tokin *et al.*, 2021 ▸). For both *Ls*AA9_A(f) and *Ls*AA9_A(*Ec*) oligosaccharide-binding structures, there are no considerable structural changes in inter­actions (*e.g.* protein-accessible surface or hydrogen bonding) in the Cu^2+^ to Cu^+^ transition that can explain the increased affinity. However, since the axial ligand is already lost during reduction, no penalty must be paid for the loss of this inter­action in the Cu^+^ form. Furthermore, we have shown that both photoreduction and, to an even larger extent, oligosaccharide binding increases the θ_T_ angle. Thus, the oligosaccharide-free Cu^+^ state is closer in active-site geometry to the oligosaccharide-bound state, which might contribute favourably to binding.

### 3.5. The active-site tyrosine in oligosaccharide-free and oligosaccharide-bound structures

The role of the Cu-site tyrosine has been proposed to be for protection against auto-oxidation (Paradisi *et al.*, 2019 ▸; Singh *et al.*, 2019 ▸). Recently, deprotonation of tyrosine as part of the mechanism was investigated through theoretical calculations and spectroscopic measurements to elucidate the formation of the LPMO reaction inter­mediates *cis*/*trans*-[Tyr-O^**.**^–Cu^2+^–OH]^+^ (McEvoy *et al.*, 2021 ▸). In the unbound state, the formation of Tyr inter­mediates is associated with a reduction in the Tyr-O to Cu distance of 0.27–0.47 Å (McEvoy *et al.*, 2021 ▸). Thus, though a shortening has been reported from a com­parison of experimental structures (Frandsen *et al.*, 2016 ▸), it was important to confirm it in this more systematic study.

**Figure 6:**
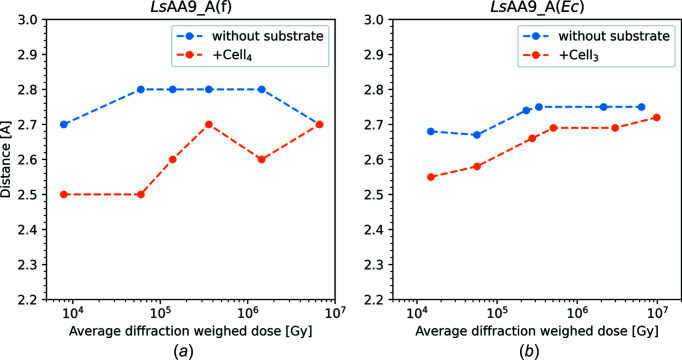
The measured Cu—Tyr-O distance over the Cu^2+^ to Cu^+^ transition for *Ls*AA9_A(f) and *Ls*AA9_A(*Ec*). A slight increase in the distance is found over the transition and a more significant reduction in the distances for all doses is found once substrate is bound. This is further presented for the low-dose structures in Movie S6.

Here, for both *Ls*AA9_A variants, the distance between Tyr164 and Cu increases by 0.1 Å with increasing radiation dose, while for *Ta*AA9_A, the distance decreases by the same value. These differences may not be significant. However, confirming the previous crystallographic studies (Frandsen *et al.*, 2016 ▸; Simmons *et al.*, 2017 ▸), the distance from Tyr164-O to Cu is shorter (0.1–0.2 Å) for all the *Ls*AA9_A(f)-Cell_4_ and *Ls*AA9_A(*Ec*)-Cell_3_ structures com­pared to the corresponding unbound structures (see Fig. 6 ▸). This difference may again not seem significant when considering only DPI-based coordinate errors (Kumar *et al.*, 2015 ▸; Murshudov *et al.*, 1997 ▸). However, there are a number of considerations suggesting that it is significant, at least for the low-dose *Ls*AA9_A Cu^2+^ structures from this and previous studies (Frandsen *et al.*, 2016 ▸), which are listed in Table S9, with the relevant distances and errors presented in Movie S6. First of all, taking each structure as an independent estimate of bond length, the average for the three saccharide-free structures is 2.71 ± 0.031 Å, while for the three saccharide-binding structures, the average is 2.50 ± 0.044 Å, indicating that the real coordinate error may be lower than estimated. Secondly, to investigate the structural changes without influence of the refinement protocol and the presence of the saccharide in the model, we calculated difference maps where only rigid-body refinement was applied to a model of a sac­charide-free structure (protein and Cu only, as a single rigid body to maintain a fixed distance) against data for a sac­charide-binding structure. Maps calculated for three such pairs show clearly that the data demand the copper–Tyr distance to be shortened (Fig. S15). The reduction in distance between Tyr-O and Cu is also reproduced qualitatively in the QM/MM-optimized structures of *Ls*AA9_A, where the distance was found to decrease by 0.2–0.3 Å upon *Ls*AA9_A binding the substrate (Theibich *et al.*, 2021 ▸). While the role of tyrosine remains unclear, the shortening of the Tyr-O to Cu bond has been shown here to be reproducible for *Ls*AA9_A.

## 4. Conclusion

For metalloproteins, radiation damage in the form of photoreduction is common, and balancing X-ray dose and diffraction intensity is crucial. For LPMOs, photoreduction of the central Cu atom is difficult to avoid in a diffraction experiment, but may reveal details for the first step of the LPMO reaction.

We recommend that researchers wishing to investigate the LPMO Cu^2+^ state plan their experiments to limit the total dose to a few kGy or less if possible and, if necessary, to use helical data collections to reduce the dose. A dose in the MGy range should be used to catch Cu^+^ states, or possibly trigger the LPMO reaction. Similarly, we were successful in collecting data on chemically reduced crystals which support a Cu^+^ state.

Through our photoreduction study we have monitored various geometrical parameters, which are indicative of the two LPMO Cu states. As the observed changes in distance to protein ligands are small, the best structural diagnostics for the Cu^2+^ to Cu^+^ transition in these (and probably other octa­hedrally coordinated Cu sites) are the θ_3_ and θ_T_ values, where values lower than 170° and higher than 3°, respectively, are strong indicators of a reduced site. Additional indicators may be found in the angles θ_H–H_ and θ_H1_, which appear lower for a reduced site, and θ_HN_, which is slightly higher for the Cu^+^ state, although the absolute values are inconsistent between the investigated proteins. Non-protein ligands may also be helpful in identifying the Cu state, as we and others have demonstrated, and the final T-shaped geometry of the His-brace seems to be a defining feature of LPMO Cu^+^ sites. The exogenous ligands can be affected by the closeness of other mol­ecules present in the active site, which must be considered when inter­preting structural data. Thus, when a single-crystal X-ray experiment is performed, these values alone cannot firmly assign a structure as Cu^2+^ or Cu^+^.

Upon binding of saccharide substrate, the Cu–Tyr distance in AA9s is found consistently to be shortened, regardless of the Cu state. Based on several independently determined structures and an analysis of difference electron-density maps, we find this small shortening of ∼0.2 Å significant for the Cu^2+^ structures of *Ls*AA9_A. With previous indications that the active-site Tyr is important for the mechanism, the experimental structures now available at both Cu stages could allow further com­putational exploration. Correlated to the Cu–Tyr distance changes, while in Cu^2+^ saccharide-free structures the equatorial ligands and Cu are close to being coplanar, saccharide binding induces clear deviation from planarity, as seen in changes of the θ_T_ angle. As the reduction of Cu also induces geometric changes to the Cu site, including an increase in the θ_T_ angle, the observed greater affinity of Cu^+^ LPMOs for poly- and oligosaccharides may be partly explained by being closer structurally to the oligosaccharide-bound form.

## 5. Abbreviations

AA: auxiliary activity; ASC: ascorbic acid; ax: Axial; CAZy: Carbohydrate-Active enZymes database; Cell_3_: cellotriose; Cell_4_: cello­tetra­ose; EndoH: endoglycosidase-H; Eq: equatorial; LPMO: lytic polysaccharide monooxygenase; *Ls*AA9_A(*Ec*): *Ls*AA9_A produced in *E. coli*; *Ls*AA9_A(f): *Ls*AA9_A produced in *A. oryzae*; *Ls*AA9_A: family AA9 LPMOs from *Lentinus similis*; MX: macromolecular crystallography; QM/MM: quantum mechanics/mol­ecular mechanics; RT: room tem­per­ature; SSX: serial synchrotron crystallography; *Ta*AA9_A: family AA9 LPMOs from *Thermoascus auranti­acus.*

## Supplementary Material

PDB reference: *Ls*AA9_A(f), 7pxi

PDB reference: 7pxj

PDB reference: 7pxk

PDB reference: 7pxl

PDB reference: 7pxm

PDB reference: 7pxn

PDB reference: RT, 7pxr

PDB reference: RT (synchrotron), 7pxs

PDB reference: reduced with ascorbic acid, low X-ray dose, 7pxu

PDB reference: reduced with ascorbic acid, high X-ray dose, 7pxv

PDB reference: SSX, 7pxt

PDB reference: complex with cello­tetraose, 7pyd

PDB reference: 7pye

PDB reference: 7pyf

PDB reference: 7pyg

PDB reference: 7pyh

PDB reference: 7pyi

PDB reference: *Ls*AA9_A(*Ec*), 7pyl

PDB reference: 7pym

PDB reference: 7pyn

PDB reference: 7pyo

PDB reference: 7pyp

PDB reference: 7pyq

PDB reference: 7pqr

PDB reference: complex with cellotriose, 7pyu

PDB reference: 7pyw

PDB reference: 7pyx

PDB reference: 7pyy

PDB reference: 7pyz

PDB reference: 7pz0

PDB reference: complex with cellotetraose, 7pxw

PDB reference: *Ta*AA9_A, 7pz3

PDB reference: 7pz4

PDB reference: 7pz5

PDB reference: 7pz6

PDB reference: 7pz7

PDB reference: 7pz8

Movie S1. DOI: 10.1107/S2052252522007175/mf5061sup1.mp4

Movie S2. DOI: 10.1107/S2052252522007175/mf5061sup2.mp4

Movie S3. DOI: 10.1107/S2052252522007175/mf5061sup3.mp4

Movie S4. DOI: 10.1107/S2052252522007175/mf5061sup4.mp4

Movie S5. DOI: 10.1107/S2052252522007175/mf5061sup5.mp4

Movie S6. DOI: 10.1107/S2052252522007175/mf5061sup6.mp4

Additional figures and tables. DOI: 10.1107/S2052252522007175/mf5061sup7.pdf

Raw data archive: https://doi.org/10.17894/ucph.354e520a-e193-41a3-ac3f-87259db10fda

## Figures and Tables

**Table 1:** The final data sets derived from data collected at increasing photon flux on *Ls*AA9_A(f) crystals The 7.88 × 10^3^ Gy data set consists of the first 45° of the collection at 10^10^ photons/s. The 5.99 × 10^4^ Gy data set consists of the last 45° of the collection at 10^10^ photons/s. The 1.39 × 10^5^ Gy data set consists of the first 45° of the 10^11^ photons/s collection. The 3.60 × 10^5^ Gy data sets consists of images in the range 100–180° of the 10^11^ photons/s collection. The 1.45 × 10^6^ Gy data sets consists of the first 45° of the 10^12^ photons/s collection. Finally, the 6.65 × 10^6^ Gy data set is from the last 45° of the 10^12^ photons/s collection.

Photon flux	10^10^ photons/s		10^11^ photons/s		10^12^ photons/s	
Data range used (°)	0–45	315–360	0–45	100–180	0–45	315–360
X-ray dose (Gy)	7.88 × 10^3^	5.99 × 10^4^	1.39 × 10^5^	3.60 × 10^5^	1.45 × 10^6^	6.65 × 10^6^

**Table 2:** A brief overview of the proteins and crystals used in this study

Protein	PDB entry	Post-translational modification	Oligosaccharide ligand	Occupied binding subsites	Mother liquor com­position	Protein buffer	Space group	No. of molecules in asymmetric unit	Maximum resolution in this study (Å)	Non-H_2_O exogenous ligands
*Ls*AA9_A *Ls*AA9_A(f)	7pxi	His1 N^ɛ2^ methyl­ation; N-glycosyl­ation on Asn33			3.3 *M* NaCl, 0.1 *M* citric acid pH 3.5	50 m*M* sodium acetate pH 5.5	*P*4_1_32	1	1.30	
	7pxj									
	7pxk									
	7pxl									
	7pxm									
	7pxn									
										
*Ls*AA9_A *Ls*AA9_A(f)	7pyd	His1 N^ɛ2^ methyl­ation; N-glycosyl­ation on Asn33	1.0 *M* cellotetra­ose	−2 − 1 + 1 + 2	3.3 *M* NaCl, 0.1 *M* citric acid pH 3.5	50 m*M* sodium acetate pH 5.5	*P*4_1_32	1	1.90	Cl^−^ †
	7pye									
	7pyf									
	7pyg									
	7pyh									
	7pyi									
										
*Ls*AA9_A *Ls*AA9_A(*Ec*)	7pyl 7pym 7pyn 7pyo 7pyp 7pyq	‡			2.3 *M* (NH_4_)_2_SO_4_, 0.1 *M* sodium acetate pH 4.5	25 m*M* Bis-Tris pH 5.8, 175 m*M* NaCl	*P*4_1_	1	1.30	SO_4_ §
										
*Ls*AA9_A *Ls*AA9_A(*Ec*)	7pyu	‡	0.5 *M* cellotriose	−1 + 1 + 2¶	2.3 *M* (NH_4_)_2_SO_4_, 0.1 *M* sodium acetate pH 4.5	25 m*M* Bis-Tris pH 5.8, 175 m*M* NaCl	*P*4_1_	1	1.20	Cl^−^ †
	7pyw									
	7pyx									
	7pyy									
	7pyz									
	7pz0									
										
*Ta*AA9_A	7pz3	His1 N^ɛ2^ methyl­ation; N-glycosyl­ation on Asn138			20 m*M* MgCl_2_, 0.1 *M* HEPES pH 7.5, 22%(*w*/*v*) polyacrylic acid 5100 sodium salt	50 m*M* sodium acetate pH 5.5	*P*2_1_	1	1.40	Acrylic acid§
	7pz4									
	7pz5									
	7pz6									
	7pz7									
	7pz8									

† Cl present from crystallization conditions is often found in substrate-bound structures and is expected to inhibit the position binding either O_2_ or H_2_O_2_ (Frandsen *et al.*, 2016 ▸).

‡ *Ls*AA9_A(*Ec*) is lacking the N-terminal His methyl­ation, as expected from the bacterial expression (Fig. S1)

§ Not coordinating directly to Cu. Present from crystallization conditions.

¶ Cellotriose is also bound in a pocket at β-sheet 4 (Fig. S2). Binding in this pocket is not believed to have any biological relevance.

**Table 3:** Structural parameters within the LPMO Cu site The lowest and highest X-ray dose structures are listed, together with additional reference structures. Distances (between the indicated atom and Cu) and angles were measured in *Coot* (Emsley & Cowtan, 2004 ▸) and using the *Biopython* module (Cock *et al.*, 2009 ▸). In cases where a ligand atom is modelled in a double conformation, the distance is an average weighted by the occupancy of the atom. Further structural parameters for all structures are presented in Tables S7 and S8. Res = high-resolution limit. Distances are measured from Cu to the indicated atom. θ_1_, θ_2_, θ_3_ and θ_T_ (°) are defined in Vu & Ngo (2018 ▸). The lowest/highest/average distance and θ_1_, θ_2_, θ_3_ and θ_T_ (°) for Cu^2+^/Cu^+^ structures are from Vu & Ngo (2018 ▸). ECR = estimated coordinate error based on the *R* value; value extracted from the *REFMAC5* output PDB file.

	PDB entry	Dose (Gy)	Res. (Å)	*R*_work_/*R*_free_ (%)	N^δ1^ (Å)	N_Am_ (Å)	N^ɛ2^ (Å)	O_Tyr_ (Å)	Eq (Å)	Ax (Å)	θ_1_, θ_2_, θ_3_ (°)	θ_T_ (°)	ECR (Å)
*Ls*AA9_A(f) (RT)	7pxr		1.80	14.67/16.62	1.9	2.2	2.0	2.8	2.2	2.8	88.6, 92.9, 176.6	3.3	0.081
*Ls*AA9_A(f) (RT-sync)	7pxs	1.91 × 10^3^	1.90	16.94/19.56	1.9	2.2	2.0	2.8	2.2	2.6	92.7, 94.7, 170.5	5.9	0.106
*Ls*AA9_A(f) (Ascorbic acid)	7pxu	2.08 × 10^3^	1.80	19.52/22.65	1.9	2.3	2.0	2.9	4.0	3.7	94.8, 96.3, 168.4	3.4	0.119
	7pxv	1.70 × 10^7^	1.50	18.74/20.70	1.8	2.3	2.0	2.8	4.0	3.5	93.2, 97.4, 168.6	4.4	0.063
*Ls*AA9_A(f)	7pxi	7.88 × 10^3^	1.57	18.30/20.99	1.9	2.2	2.0	2.7	2.2	2.7	92.9, 89.6, 177.1	1.5	0.080
	7pxn	6.65 × 10^6^	1.65	20.35/22.18	1.9	2.3	2.0	2.7	3.3	3.3	91.7, 99.7, 167.5	5.1	0.057
*Ls*AA9_A(*Ec*)	7p­yl	1.49 × 10^4^	1.70	14.83/18.83	2.0	2.1	2.0	2.7	1.9	2.6	91.8, 94.9, 172.6	3.3	0.094
	7pyq	6.35 × 10^6^	1.60	14.42/16.78	2.0	2.2	2.0	2.8		3.8	93.3, 102.9, 162.0	7.8	0.069
*Ls*AA9_A(f) (SSX)	7pxt	7.02 × 10^4^	2.40	18.67/24.76	1.9	2.3	1.9	2.7	3.9	2.9	84.9, 99.6, 171.5	7.1	0.319
*Ta*AA9_A	7pz3	5.37 × 10^3^	1.90	20.81/25.63	1.9	2.2	2.1	3.0	2.2	2.0	94.0, 91.4, 174.6	−1	0.207
	7pz8	3.12 × 10^6^	1.40	15.12/16.89	1.9	2.2	2.0	2.9	2.5	3.0	94.6, 96.1, 169.3	1	0.055
*Ls*AA9_A(f)-Cell_4_	7pyd	7.88 × 10^3^	2.21	22.47/28.71	2.0	2.3	2.0	2.5	2.3		96.4, 94.1, 161.1	15.6	0.246
	7pyi	6.65 × 10^6^	2.05	21.80/26.00	1.9	2.5	2.0	2.7	3.8		98.1, 110.1, 147.9	14.5	0.184
*Ls*AA9_A (*Ec*)-Cell_3_	7pyu	1.49 × 10^4^	1.40	14.19/16.22	2.0	2.1	2.0	2.6	2.3		91.3, 91.7, 169.2	10.4	0.052
	7pz0	9.81 × 10^6^	1.20	13.40/14.75	1.9	2.3	1.9	2.7	3.8		93.0, 98.8, 165.7	8.2	0.030
*Ls*AA9_A(*Ec*)-Cell_4_	7pxw	2.14 × 10^6^	1.40	11.71/15.98	2.0	2.3	2.0	2.7	3.9		92.4, 99.5, 165.3	8.5	0.047
													
Cu^2+^													
Lowest					2.1	1.9	1.9				88, 85, 168	−2.4	
Highest					2.4	2.3	2.4				103, 96, 178	4.2	
Average					2.2	2.0	2.1				93, 92, 174	0.75	
													
Cu^+^													
Lowest					2.0	1.9	1.9				93, 93, 155	−14	
Highest					2.3	2.1	2.3				99, 103, 169	16	
Average					2.2	2.0	2.0				96, 99, 163	3.9	
