# Supplementary material for: Changes in active-site geometry on X-ray photoreduction of a lytic polysaccharide monooxygenase active-site copper and saccharide binding
Source: IUCrJ. 2022 Aug 17;9(Pt 5):666–81. doi: 10.1107/S2052252522007175 (PMC9438499; doi:10.1107/S2052252522007175)
Supplement: Supplementary file 7 [file m-09-00666-sup7.pdf]

IUCrJ

Volume 9 (2022)

Supporting information for article:

Changes in active-site geometry on X-ray photoreduction of a lytic polysaccharide monooxygenase active-site copper and saccharide binding

Tobias Tandrup, Sebastian J. Muderspach, Sanchari Banerjee, Gianluca Santoni, Johan Ø. Ipsen, Cristina Hernández-Rollán, Morten H. H. Nørholm, Katja S. Johansen, Flora Meilleur and Leila Lo Leggio

Supplementary Figures

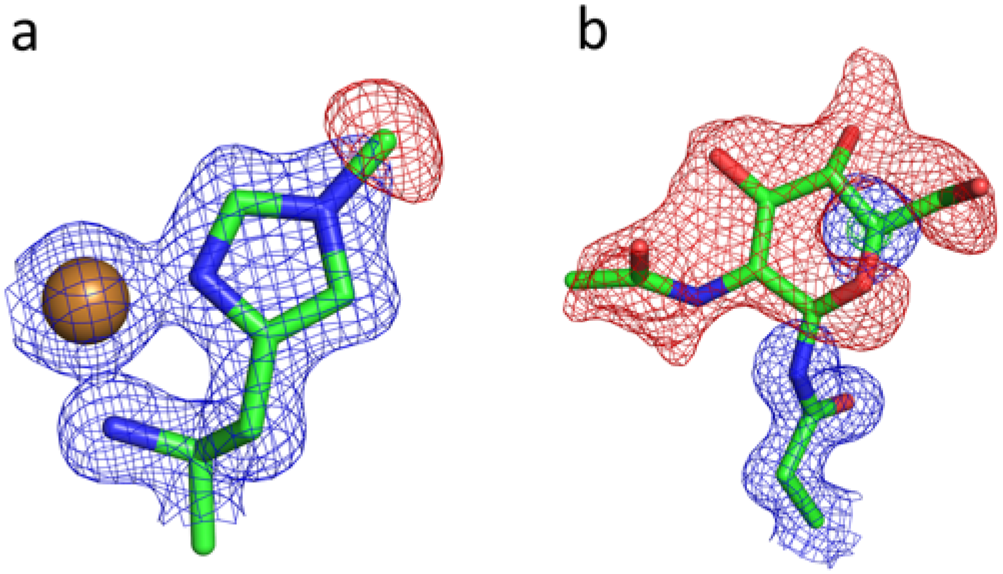

Figure S1 The main differences between the fungal and bacterially expressed *Ls*AA9_A. The difference density map (F_o-F_c) at contour level ±3.0 σ shown in green and red for positive and negative difference density respectively. The electron density map ($2F_o$-F_c) is shown as blue mesh and is contoured at 1.0 σ. (a) The N-terminal histidine of *Ls*AA9_A is shown before the methylation was deleted in the refinement process. The red density at the methylation indicates that the model is oversaturated at that location and the methylation should not be there. (b) The glycosylation site at Asn33 exhibit a large negative density, indicating absence of glycosylation in this structure. A positive difference density is seen at the C5 position of the shown N-Acetylglucosamine molecule, where a water molecule should be modelled. PDB code: 7PQR.

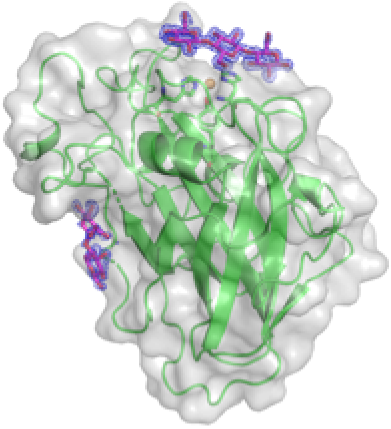

Figure S2 Substrate binding of *Ls*AA9_A(*Ec*) soaked in cellotriose. The substrates are shown as magenta sticks and *Ls*AA9_A(*Ec*) is depicted as the green cartoon. Cellotriose can be seen bound at the histidine brace (top), while a secondary binding site is found at the opposite side of the enzyme. $2F_o$-F_c electron density map shown in blue mesh at 1.0 σ contour level. PDB code: 7PYU.

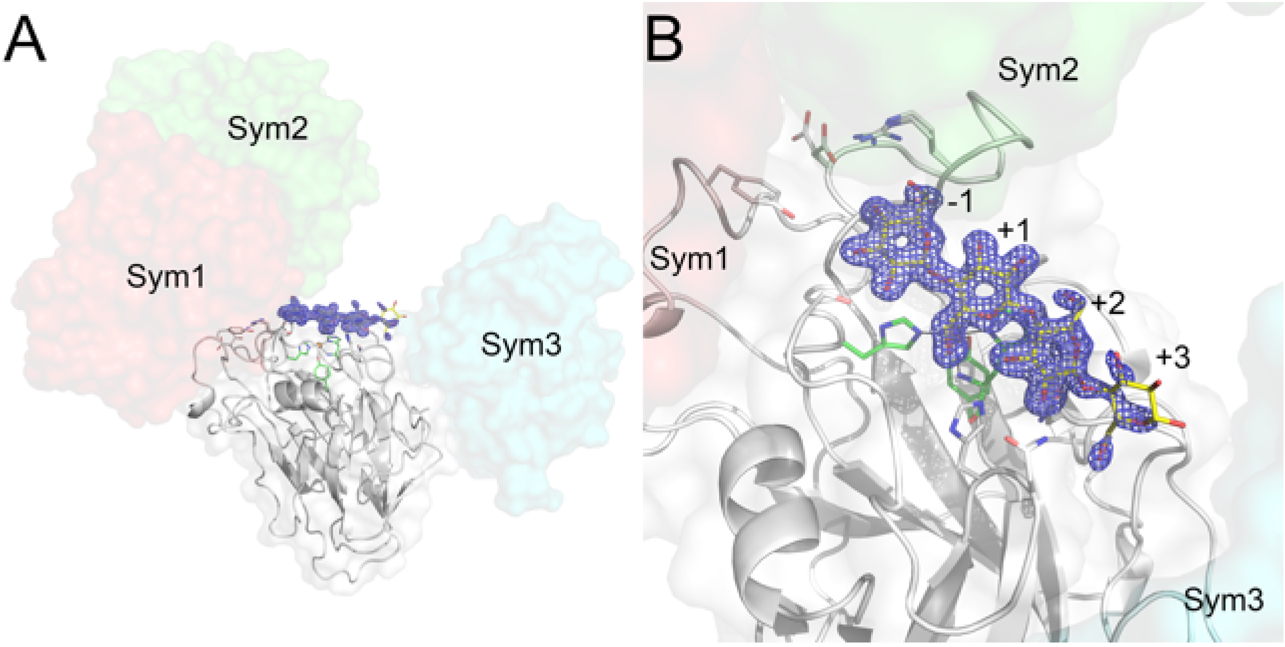

Figure S3 Substrate binding of *Ls*AA9_A(*Ec*) soaked in cellotetraose. A) An overview of the overall structure with symmetry related molecules. B) A zoomed in view of the active site binding surface. The substrate is shown in yellow sticks with $2F_o$-F_c electron density map shown in blue mesh at 1.0 σ contour level. Cellotetraose is bound from subsite -1 to +3, and not from -2 to +2 as has been seen for *Ls*AA9_A-Cell$_4$ (PDB 6YDG) (Tandrup *et al.*, 2020). This is due to symmetry related molecules (Sym1 and Sym2 in red and green surfaces) occluding the -2 subsite. Symmetry related molecule Sym3 is shown in blue similarly prevents longer substrates from making meaningful interactions at further positive subsites. PDB code: 7PXW.

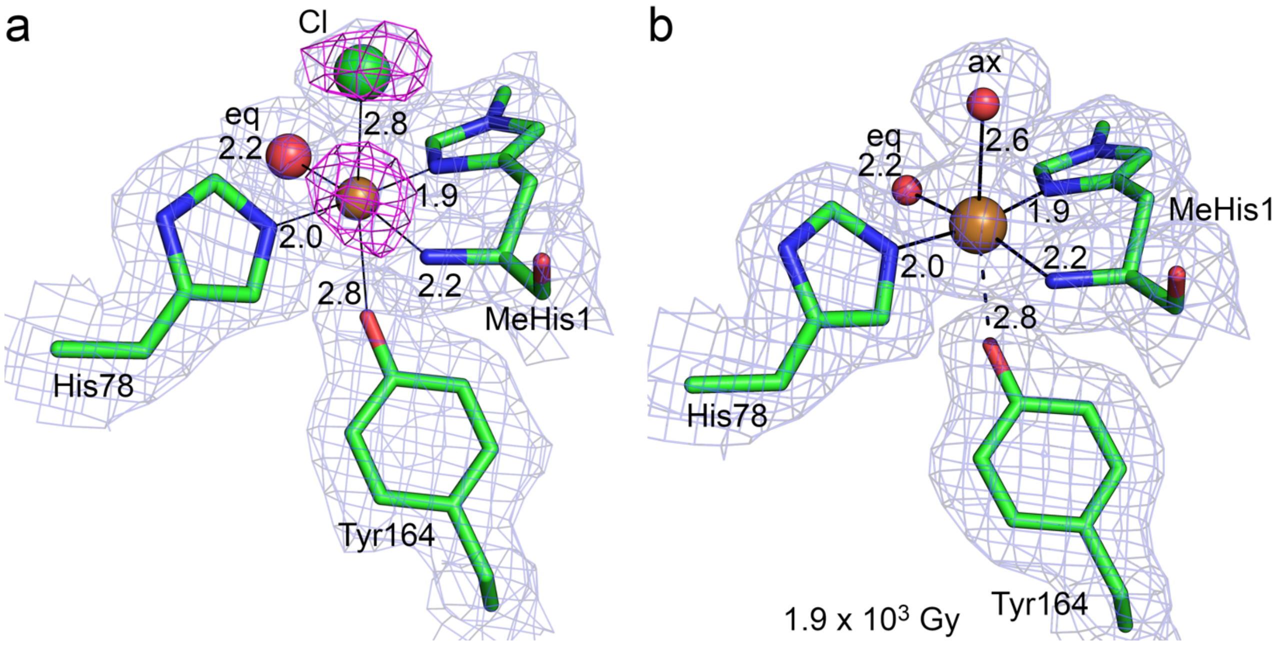

Figure S4 Active site of *Ls*AA9_A(f) at room temperature. Data were collected at an in-house diffractometer (a) and at a synchrotron (b). The axial position in the structure from in-house data is occupied by a Cl^- ion, and by the more commonly observed water in the structure from synchrotron data. Anomalous difference map (purple mesh) is shown at 4.0 σ contour level. Active site distances are shown in Å and listed in Table 3 and Supplementary Table 7. $2F_O$-F_C map (blue mesh) is shown at 1.0 σ contour level. PDB codes: 7PXR (a), 7PXS (b).

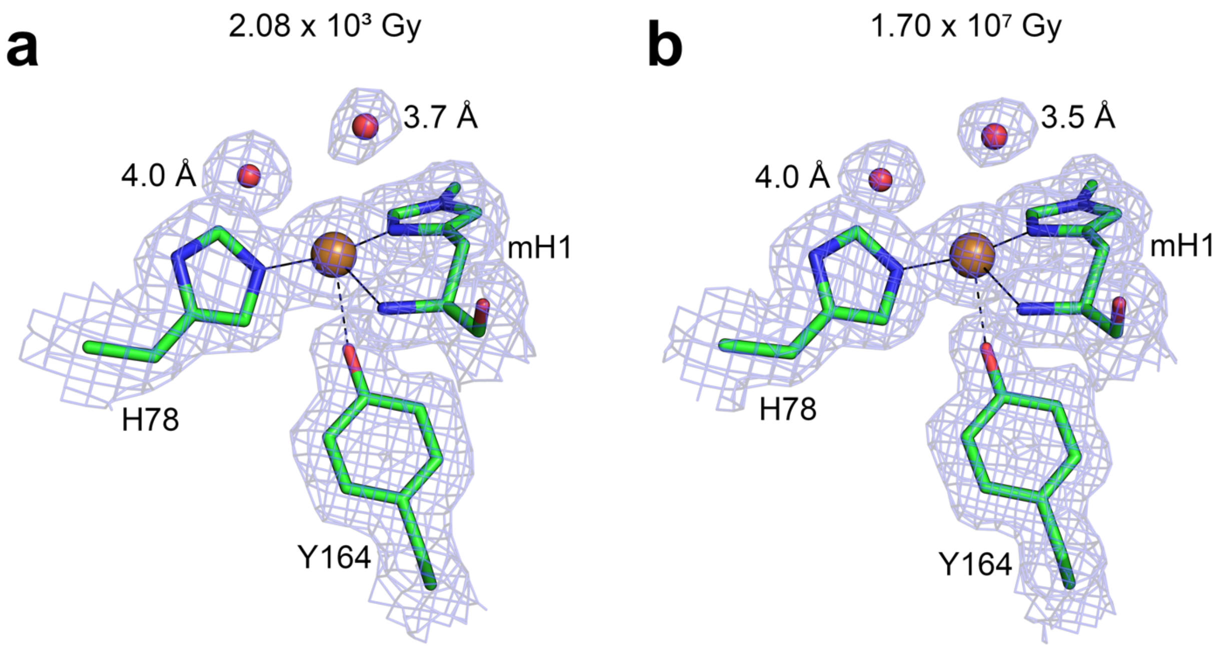

Figure S5 Active site of *Ls*AA9_A structure solved from crystal soaked in ascorbic acid. The two structures have a received X-ray dose of a) 2.08×10^3 Gy and b) 1.70×10^7 Gy. Active site distances in both structures agree with a fully reduced Cu, similar to what is observed for the 6.65×10^6 Gy structure of native *Ls*AA9_A(f) presented in Figure 2b. Active site distances are listed in Table 3 and Supplementary Table 7. $2F_O$-F_C map (blue mesh) is shown at 0.8 σ contour level in panel a) and 1.0 σ contour level in b). Distances indicated in Å are given from the water molecules to the Cu. PDB codes: 7PXU (a), 7PXV (b).

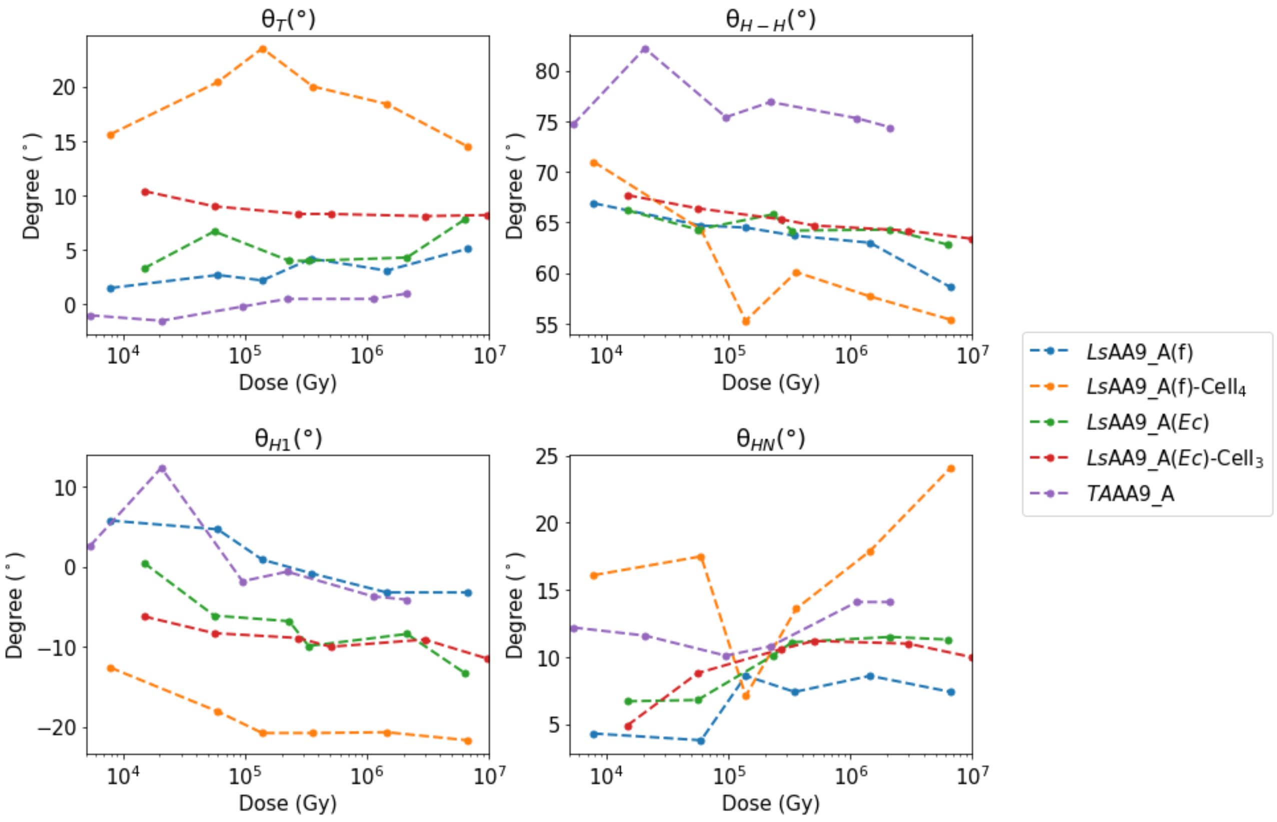

Figure S6 Active site angles as a function of X-ray dose. The defined angles (see Figure 1) of the AA9 Cu site are recorded for *Ls*AA9_A(f) and *Ls*AA9_A(*Ec*), with and without substrate, and *Ta*AA9_A. Values are listed in Table 3 and Supplementary Tables 7 and 8.

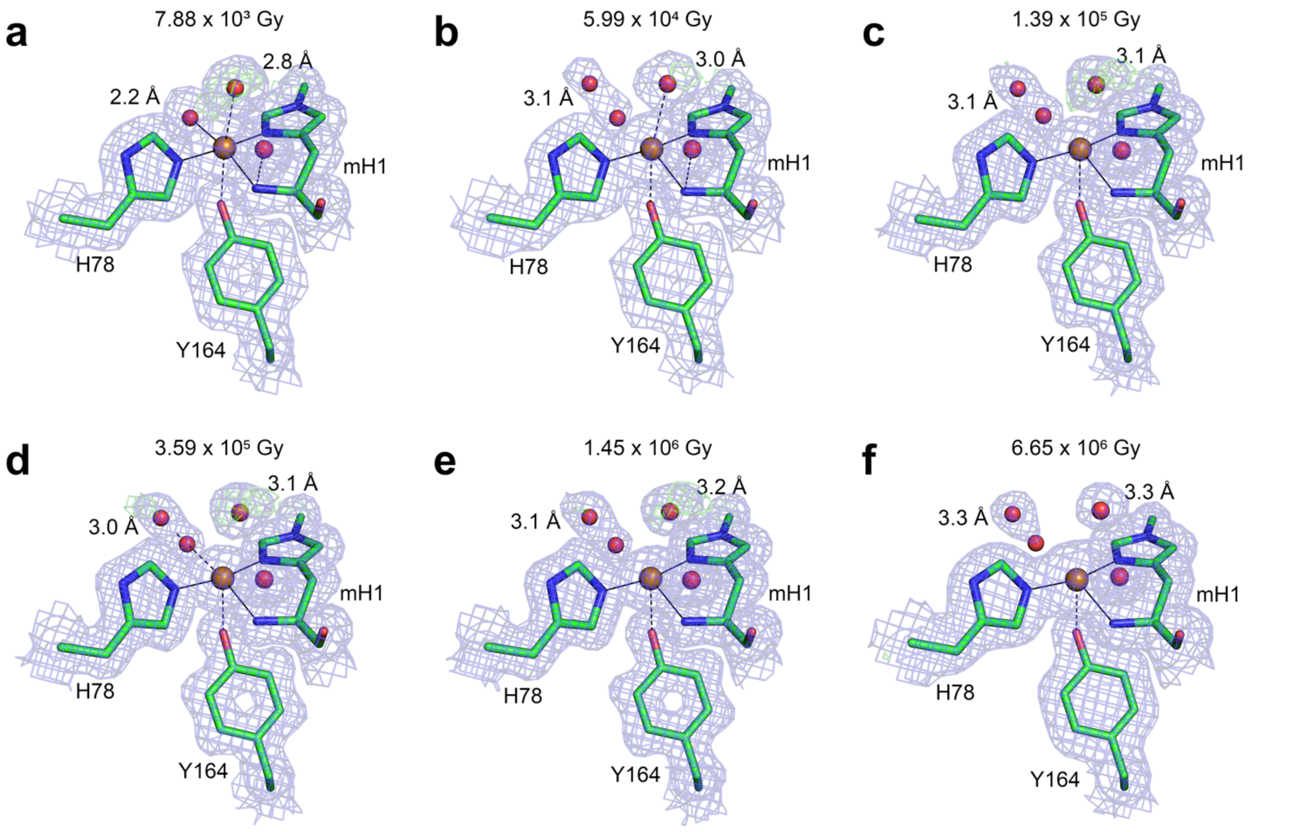

Figure S7 Active site of fungally expressed *Ls*AA9_A at increasing X-ray doses. Active site distances are presented in Supplementary Figure 9 and listed in Table 3 and Supplementary Table 7. At increasing X-ray dose both the axial and equatorial water molecules (red spheres) increase their distance to the Cu (orange sphere). In panels b-f) the equatorial water molecule has been modeled in a double conformation. For all panels the $2F_O$-F_C map (blue mesh) is shown at 1.0 σ contour level and difference map in green/red mesh at +/-3.0 σ contour level. An animation of the transition between these structures is available as Supplementary Movie 1. PDB codes: 7PXI (a), 7PXJ (b), 7PXK (c), 7PXL (d), 7PXM (e), 7PXN (f).

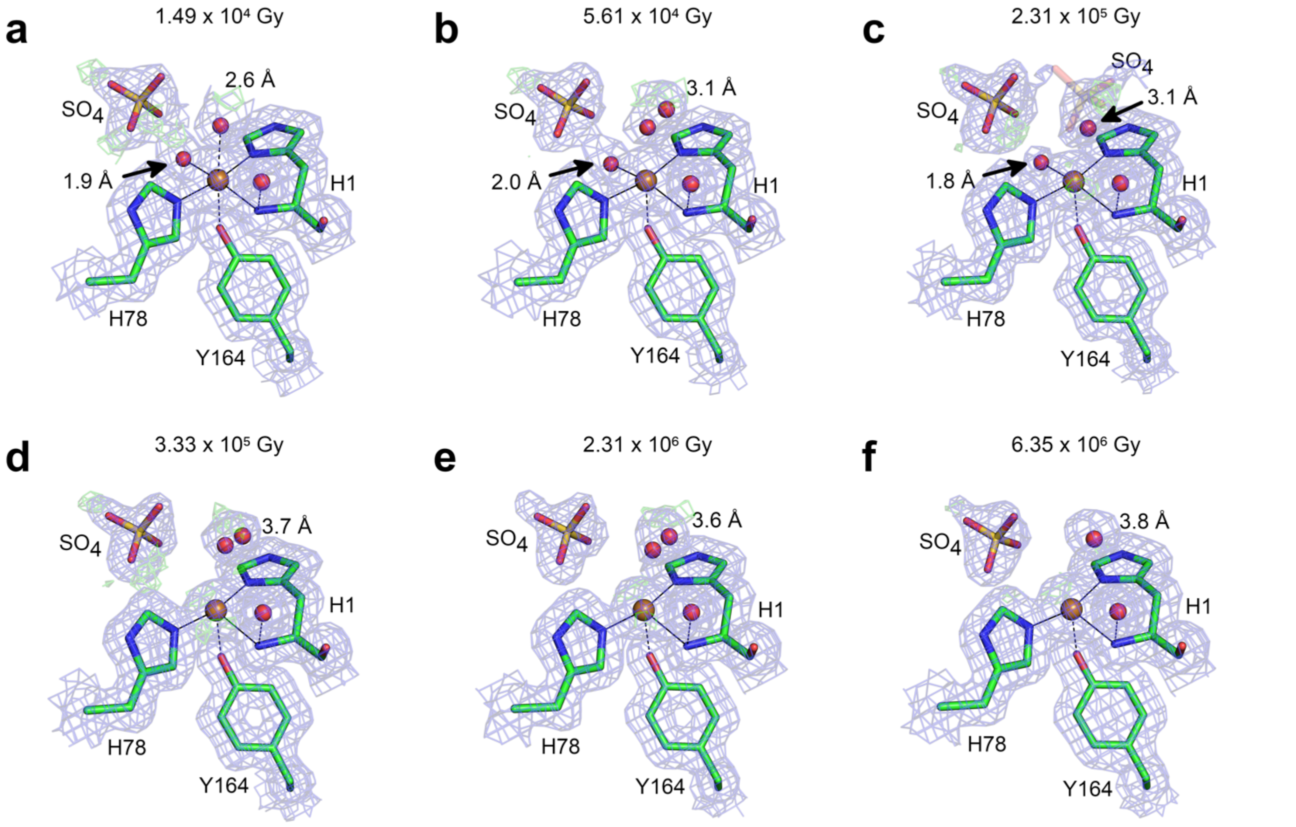

Figure S8 Active site of *Ls*AA9_A(*Ec*) at increasing X-ray doses. Active site distances are presented in Supplementary Figure 9 and listed in Table 3 and Supplementary Table 7. The distance between Cu (orange sphere) and the axial water molecule (red sphere) increases with increasing X-ray dose. In panels b,d,e) the axial water molecule has been modeled in a double conformation. On the opposite side of the equatorial water molecule with respect to the Cu a sulfate has been modeled. The distance between the nearest atom (SO_4-O1) and Cu is 4.3 Å (a). As the X-ray dose increase, this distance is reduced to 3.9 Å for the highest dose *Ls*AA9_A(*Ec*) structure (f). In panel c) it was possible to model a partially occupied sulfate (transparent). In panel d-f) no equatorial water molecule has been modeled, as no supporting electron density was available at this position. For all panels the $2F_O$-F_C map (blue mesh) is shown at 1.0 σ contour level, and difference map in green/red mesh at +/-3.0 σ contour level. An animation of the transition between these structures is available as Supplementary Movie 2. PDB codes: 7PYL (a), 7PYM (b), 7PYN (c), 7PYO (d), 7PYP (e), 7PYQ (f).

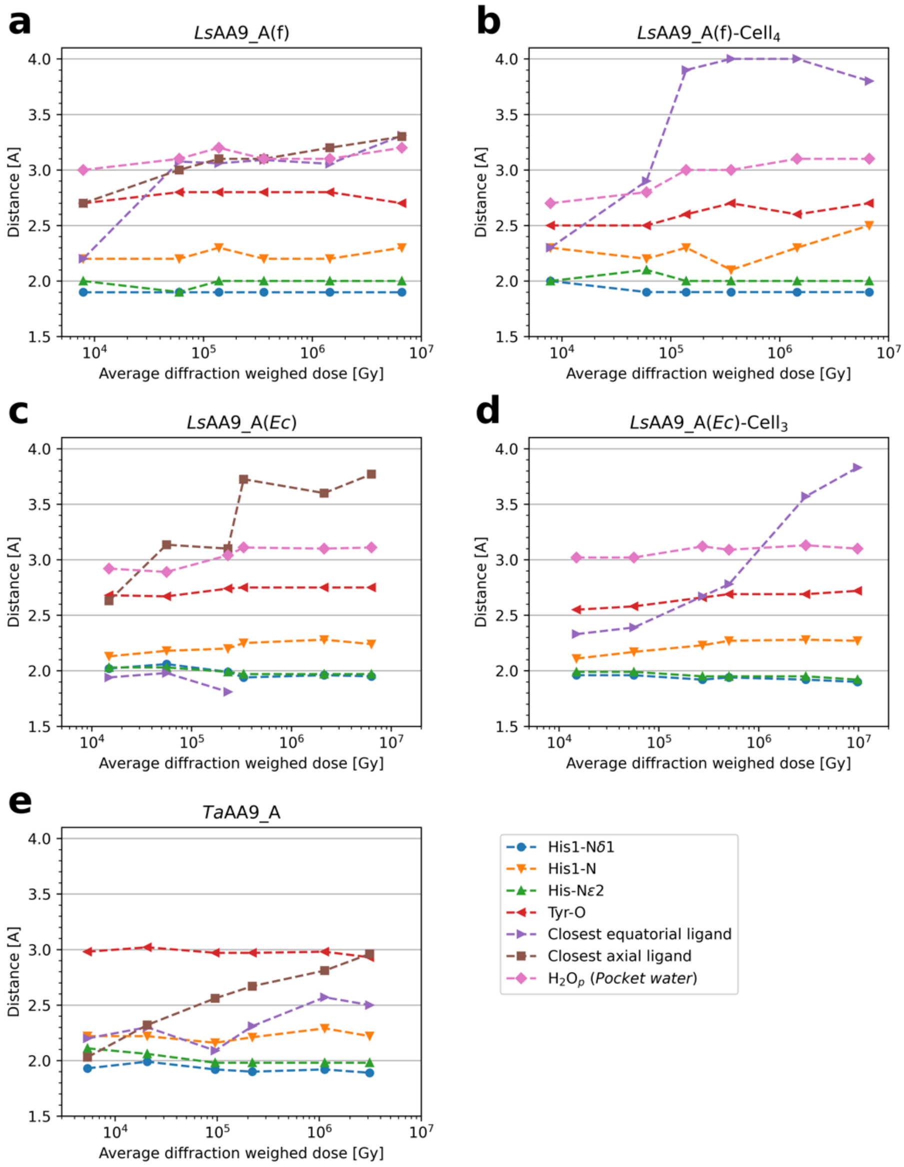

Figure S9 Measured distances as a function of average diffraction weighed radiation dose. Distances are measured from the Cu-atom to the indicated atoms. For H_2O_p, the distance is measured between the N-terminal nitrogen-atom and the oxygen atom of the nearest water molecule. All distances are listed in Supplementary Table 7.

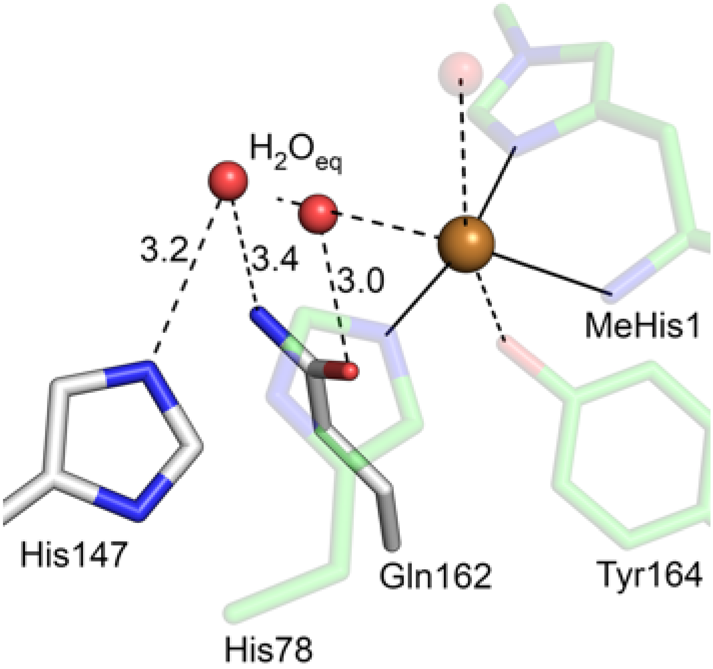

Figure S10 Secondary coordination sphere of *Ls*AA9_A at 6.65 × 10^6 Gy. The equatorial water (H_2O_{eq}), modeled in a double conformation, is positioned far from the Cu ion and may instead be H-bonded to either Gln162 or His147. Indicated distances in Å. PDB code: 7PXN.

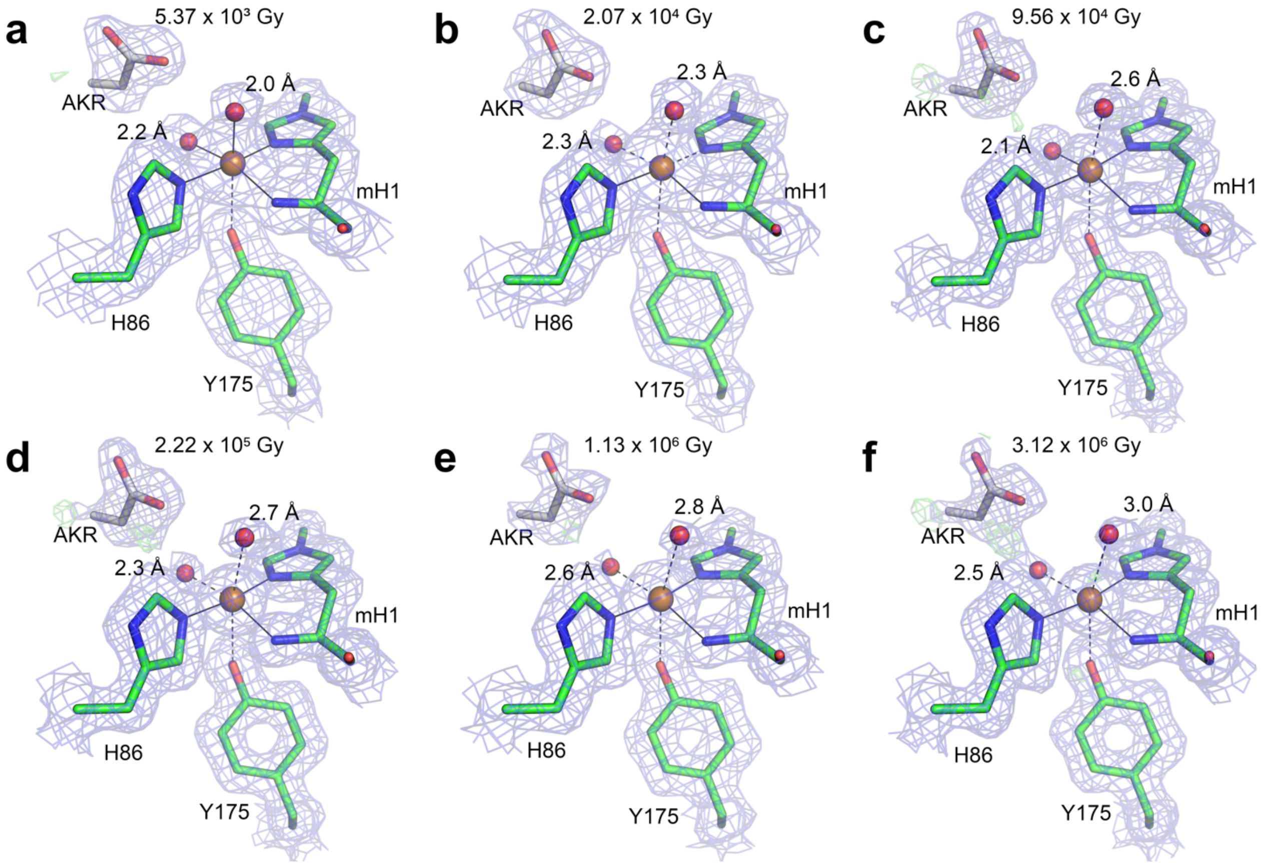

Figure S11 Active site of *Ta*AA9_A at increasing X-ray doses. Active site distances are presented in Supplementary Figure 9 and listed in Table 3 and Supplementary Table 7. With increasing X-ray dose both the equatorial and axial water molecule increase their distance to Cu. Most notably the axial water molecule, as large distances at the equatorial position is consistently prevented by polyacrylic acid, modeled as acrylic acid. Close to the axial position is also a HEPES molecule with 60 % occupancy, though not shown here for clarity. For all panels the $2F_O$-F_C map (blue mesh) is shown at 1.0 σ contour level, and difference map in green/red mesh at +/-3.0 σ contour level. An animation of the transition between these structures is available as Supplementary Movie 3. PDB codes: 7PZ3 (a), 7PZ4 (b), 7PZ5 (c), 7PZ6 (d), 7PZ7 (e), 7PZ8 (f).

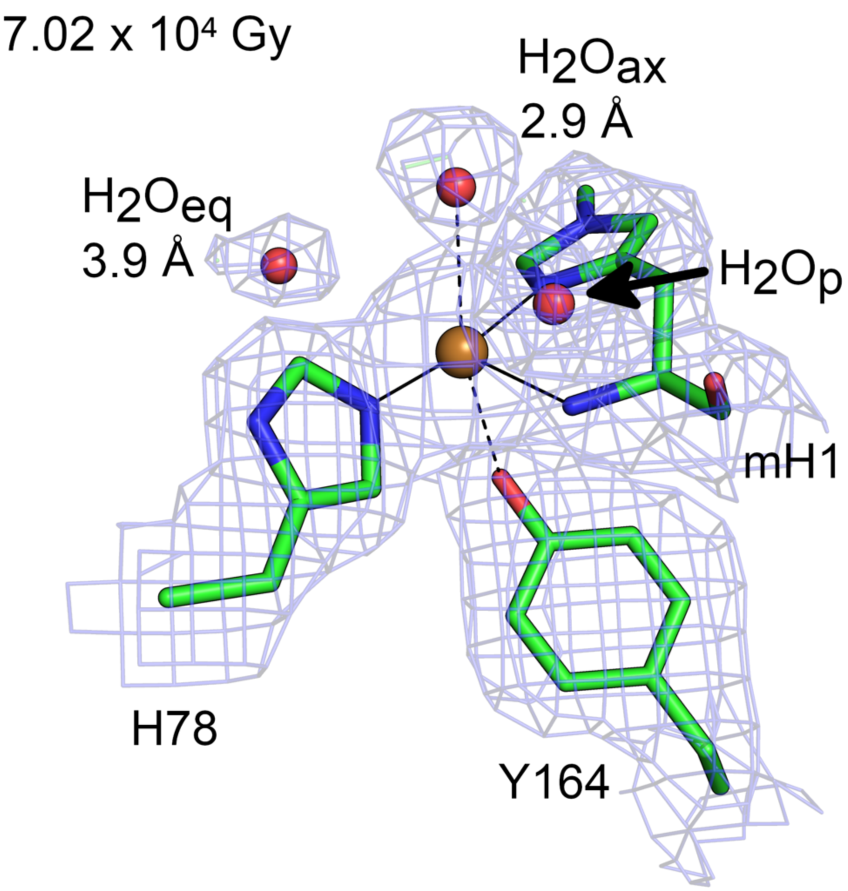

Figure S12 Active site of *Ls*AA9_A structure solved by serial synchrotron crystallography. The structure was solved from 13 crystals, each estimated to have received an X-ray dose of 7.02 × 10^4 Gy. Active site distances are to some extent comparable to the 5.99 × 10^4 Gy native *Ls*AA9_A(f) structure presented in Figure 2b. Active site distances listed in Table 3 and Supplementary Table 7. $2F_O$-F_C map (blue mesh) is shown at 1.0 σ contour level. Distances in Å are measured between the water molecule and the Cu. PDB code: 7PXT.

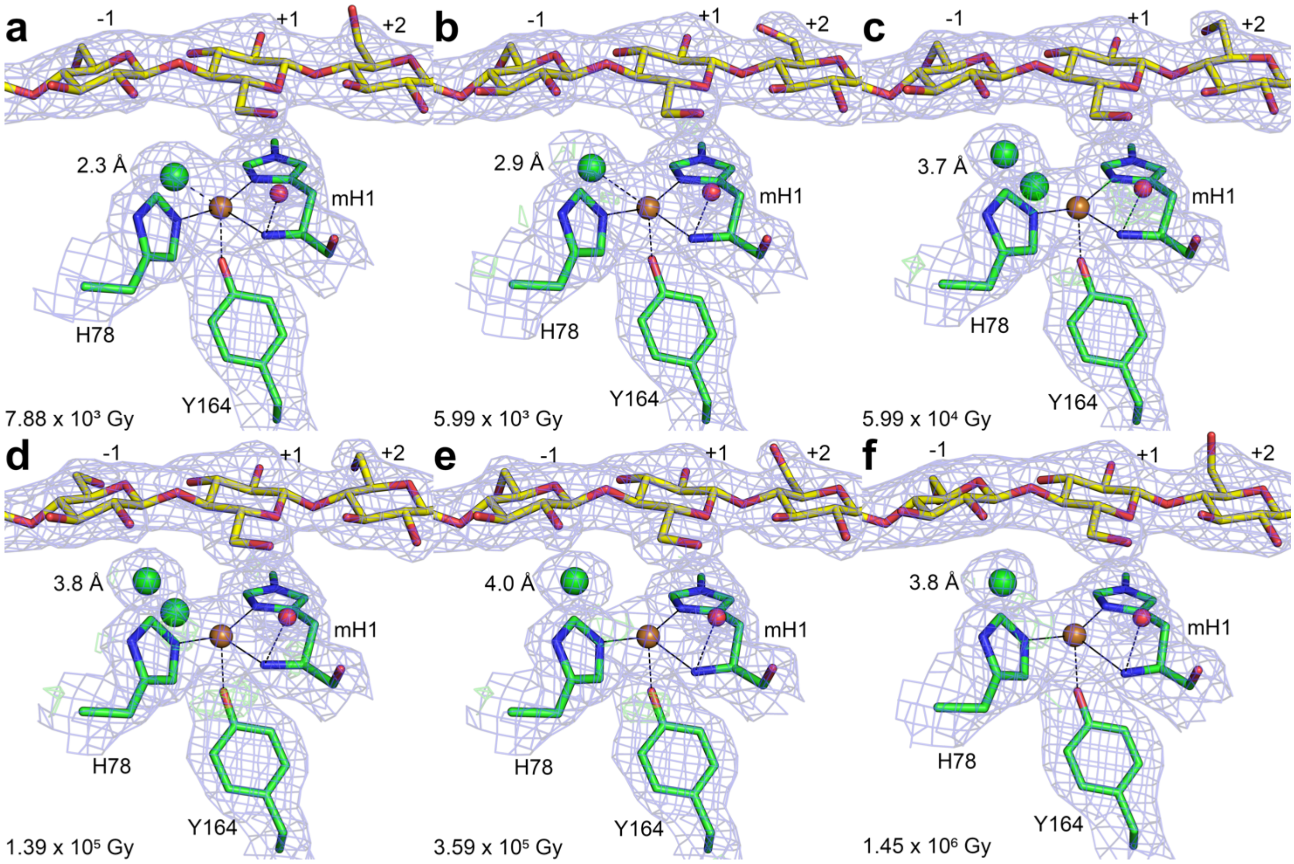

Figure S13 Active site of fungally expressed *Ls*AA9_A binding $Cell_4$ at increasing X-ray doses. Active site distances are presented Supplementary Figure 9 and listed in Table 3 and Supplementary Table 7. At increasing X-ray dose, the equatorial Cl-ion (green spheres, present from crystallization conditions) increase its distance to the Cu. Distances in Å are measured between the Cl-ion and the Cu. In panels c) and e) the equatorial water molecule has been modeled in a double conformation. The Tyr164-O to Cu distance is here in all cases shorter than for the substrate free structures presented in Supplementary Figure 7. This distance increases slightly with increasing X-ray dose (see Figure 6). For all panels the $2F_O$-F_C map (blue mesh) is shown at 1.0 σ contour level and the difference map in green/red mesh at +/-3.0 σ contour level. An animation of the transition between these structures is available as Supplementary Movie 4. PDB codes: 7PYD (a), 7PYE (b), 7PYF (c), 7PYG (d), 7PYH (e), 7PYI (f).

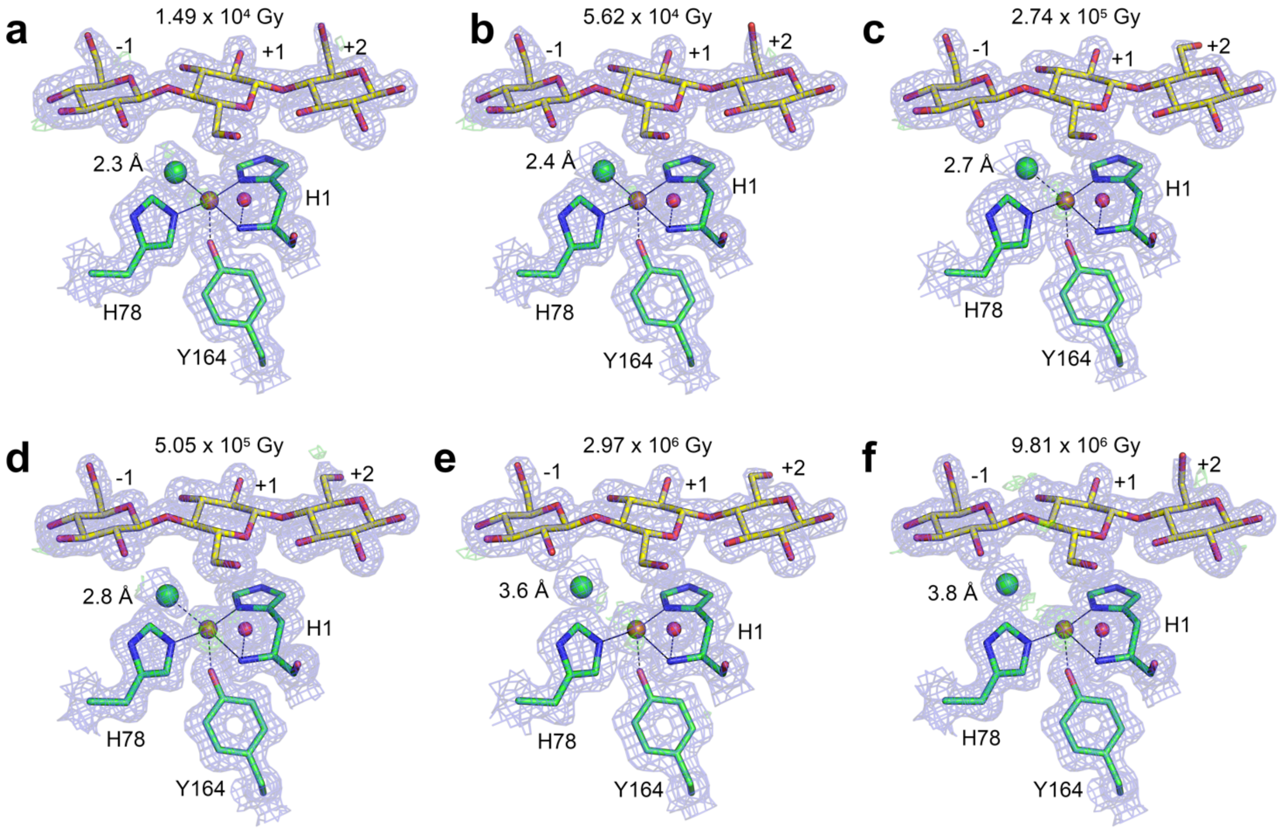

Figure S14 Active site of *Ls*AA9_A(*Ec*)-Cell$_3$ at increasing X-ray doses. Active site distances are presented in Supplementary Figure 9 and listed in Supplementary Table 7. Similar to *Ls*AA9_A(f)-Cell$_4$ no axial water molecule is present with cello-oligosaccharide bound. Cell$_3$ binds from subsite -1 to +2, as has been described previously for the fungal expressed *Ls*AA9_A (Frandsen *et al.*, 2016; Tandrup *et al.*, 2020). All panels show a well-defined Cl^- ion at the equatorial position. This Cl^- ion increase its distance to Cu with increasing X-ray dose. Distances in Å are measured between the Cl-ion and the Cu. For all panels the $2F_O$-F_C map (blue mesh) is shown at 1.0 σ contour level, and difference map in green mesh at 3.0 σ contour level. An animation of the transition between these structures is available as Supplementary Movie 5. PDB codes: 7PYU (a), 7PYW (b), 7PYX (c), 7PYY (d), 7PYZ (e), 7PZ0 (f).

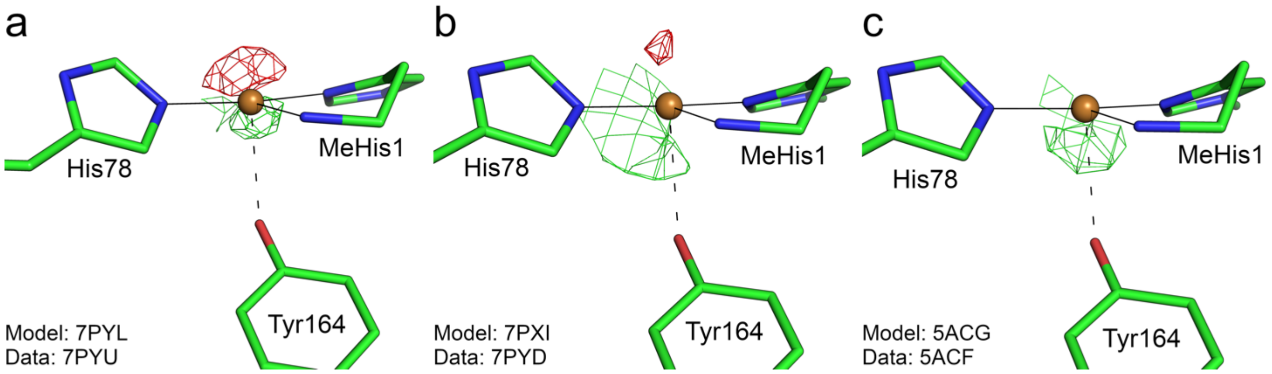

Figure S15 Difference maps demonstrating shortening of the Tyr-OH Cu distance on saccharide binding. To avoid refinement bias, difference maps were calculated using the low dose saccharide-bound data, *Ls*AA9_A(*Ec*)-Cell$_3$ (1.49 × 10^4 Gy) after rigid body refinement using the low dose saccharide-free model. A single rigid body including the protein and Cu (at fixed distance) were used for refinement and phasing (a). The difference density clearly indicate that Cu is closer to the Tyr-OH in the saccharide-bound structure. Similarly, difference maps are shown for low dose *Ls*AA9_A(f)-Cell$_4$ data (7.88 × 10^3 Gy) phased with low dose saccharide-free *Ls*AA9_A(f) model (7.88 × 10^3 Gy) (b) and previously published low dose *Ls*AA9_A-Cell$_3$ data phased with low dose *Ls*AA9_A model (Frandsen *et al.*, 2016) (c).The difference map is shown in green mesh for +3.0 σ and red mesh for -3.0 σ contour level.

Supplementary Tables

Table S1 Crystallographic data and refinement statistics for *Ls*AA9_A(f).

PDB-ID	*Ls*AA9_A(f) 7PXI	*Ls*AA9_A(f) 7PXJ	*Ls*AA9_A(f) 7PXK	*Ls*AA9_A(f) 7PXL	*Ls*AA9_A(f) 7PXM	*Ls*AA9_A(f) 7PXN
Crystal size [μm^3]	4×10^6	4×10^6	4×10^6	4×10^6	4×10^6	4×10^6
Beamline	P11	P11	P11	P11	P11	P11
Dose§ [Gy]	7.88×10^3	5.99×10^4	1.39×10^5	3.60×10^5	1.45×10^6	6.65×10^6
Wavelength [Å]	1.0332	1.0332	1.0332	1.0332	1.0332	1.0332
Flux [photons/sec]	1×10^{10}	1×10^{10}	1×10^{11}	1×10^{11}	1×10^{12}	1×10^{12}
Degrees exposed per frame	0.1	0.1	0.1	0.1	0.1	0.1
Degrees exposed	45	360	405	540	765	1080
Degrees used in processing	0-45	315-360	0-45	100-180	0-45	315-360
Exposure per frame [s]	0.1	0.1	0.1	0.1	0.1	0.1
Space group	$P4_132$	$P4_132$	$P4_132$	$P4_132$	$P4_132$	$P4_132$
Cell parameters						
(a, b, c)[Å]	124.98	124.98	124.99	124.65	125.08	125.51
(α, β, γ)[°]	90.0	90.0	90.0	90.0	90.0	90.0
Resolution [Å]	50.0-1.63 (1.67-1.63)	50.0-1.75 (1.80-1.75)	50.0-1.40 (1.44-1.40)	50.0-1.30 (1.33-1.30)	50.0-1.30 (1.33-1.30)	50.0-1.65 (1.69-1.65)
Completeness [%]	100.0 (100.0)	88.8 (92.6)	100.0 (99.9)	100.0 (100.0)	99.9 (99.6)	95.4 (97.2)
R_{meas} [%]	12.7 (134.4)	10.3 (77.2)	6.2 (141.9)	7.1 (185.2)	4.3 (108.5)	4.5 (138.5)
I/σ(I)	10.28 (1.12)	7.64 (1.27)	17.33 (1.23)	24.62 (1.46)	19.10 (1.53)	16.09 (1.02)
$CC_{1/2}$ [%]	99.8 (47.3)	99.6 (56.8)	99.9 (48.2)	100.0 (61.1)	99.9 (57.2)	99.9 (42.1)
Unique reflections	78692 (5864)	56518 (4340)	124225 (9227)	82005 (5955)	155322 (11417)	39262 (2902)
Observed reflections	404113 (28206)	109427 (8249)	630117 (43707)	1367411 (85531)	761407 (47975)	130811 (9413)
Redundancy	5.14 (4.81)	1.93 (1.90)	5.07 (4.74)	16.67 (14.36)	4.90 (4.20)	3.33 (3.24)
No. mol./ASU	1	1	1	1	1	1
R_{Work} [%]	18.30	18.31	18.68	18.73	18.46	18.02
R_{Free} [%]	20.99	21.48	20.56	19.96	20.23	20.24
Ramachandran[%]§§						
Preferred	93.1	92.7	92.7	92.7	92.7	94.0
Allowed	6.5	6.9	6.9	6.9	6.9	5.2
Outlier	0.4	0.4	0.4	0.4	0.4	0.9
Avr. B-factors[Å^2]						
Protein	18.81	18.52	17.05	16.91	17.12	34.06
Ligand / ion	-	-	-	38.37	-	-
Water	27.79	27.90	27.67	25.26	28.85	35.02
Wilson B-factor[Å^2]	27.77	27.47	24.06	23.52	22.66	30.34
No. of atoms						
Protein	1866	1866	1866	1895	1886	1901
Ligand / ion	0	0	0	3	0	0
Water	311	310	311	299	311	86
RMSD			-		-	
Bond lengths [Å]	0.011	0.010	0.013	0.014	0.015	0.012

Bond Angles [°]	1.690	1.621	1.814<br>-	1.873	1.945	1.725
Crystallization condition	3.3 M NaCl, 0.1 M citric acid pH 3.5					
Drop volume [µl]	0.5					
Ratio§§§	3:1:1					

Highest resolution shell shown in parenthesis.

§ Relative dose estimated using RADDOSE-3D (Zeldin *et al.*, 2013).

§§ Ramachandran preferred/allowed/outlier regions calculated by Rampage (Lovell *et al.*, 2003).

§§§ Protein to reservoir to water volume ratio.

Table S2 Crystallographic data and refinement statistics for *Ls*AA9_A (f)-$Cell_4$.

PDB-ID	*Ls*AA9_A-$Cell_4$<br>7PYD	*Ls*AA9_A-$Cell_4$<br>7PYE	*Ls*AA9_A-$Cell_4$<br>7PYF	*Ls*AA9_A-$Cell_4$<br>7PYG	*Ls*AA9_A-$Cell_4$<br>7PYH	*Ls*AA9_A-$Cell_4$<br>7PYI
Crystal size [μm^3]	4×10^6	4×10^6	4×10^6	4×10^6	4×10^6	4×10^6
Beamline	P11	P11	P11	P11	P11	P11
Dose§ [Gy]	7.88×10^3	5.99×10^4	1.39×10^5	3.60×10^5	1.45×10^6	6.65×10^6
Wavelength [Å]	1.0332	1.0332	1.0332	1.0332	1.0332	1.0332
Flux [photons/sec]	1×10^{10}	1×10^{10}	1×10^{11}	1×10^{11}	1×10^{12}	1×10^{12}
Degrees exposed per frame	0.1	0.1	0.1	0.1	0.1	0.1
Degrees exposed	360	360	720	720	1080	1080
Degrees used in processing	0-45	315-360	0-45	100-180	0-45	315-360
Exposure per frame [s]	0.1	0.1	0.1	0.1	0.1	0.1
Space group	$P4_132$	$P4_132$	$P4_132$	$P4_132$	$P4_132$	$P4_132$
Cell parameters						
(a, b, c)[Å]	125.64	125.64	125.62	125.67	125.72	126.02
(α, β, γ)[°]	90.0	90.0	90.0	90.0	90.0	90.0
Resolution [Å]	50.0-2.21<br>(2.27-2.21)	50.0-2.10<br>(2.15-2.10)	50.0-1.90<br>(1.95-1.90)	50.0-1.9<br>(1.95-1.9)	50.0-1.90<br>(1.95-1.90)	50.0-2.05<br>(2.10-2.05)
Completeness [%]	99.9 (100.0)	100.0 (100.0)	99.9 (100.0)	100.0 (100.0)	99.8 (99.9)	95.9 (96.7)
R_{meas} [%]	15.6 (140.0)	16.2 (251.6)	7.3 (162.4)	7.7 (183.0)	3.8 (57.9)	5.0 (144.9)
I/σ(I)	9.70 (1.24)	9.62 (0.68)	16.43 (1.08)	20.25 (1.28)	25.68 (3.17)	15.87 (1.09)
$CC_{1/2}$ [%]	99.6 (51.6)	99.8 (32.8)	99.9 (45.2)	100.0 (54.7)	100.0 (87.3)	99.9 (48.0)
Unique reflections	32082 (2405)	37414 (2785)	50437 (3753)	50564 (3763)	50544 (3756)	21166 (1596)
Observed reflections	170006 (13132)	263397 (18955)	262836 (19588)	468655 (34891)	261089 (18572)	69757 (5102)
Redundancy	5.29 (5.46)	7.04 (6.80)	5.21 (5.22)	9.26 (9.27)	5.17 (4.94)	3.29 (3.19)
No. mol./ASU	1	1	1	1	1	1
R_{Work} [%]	22.47	23.94	25.40	24.29	26.15	21.80
R_{Free} [%]	28.71	29.42	28.68	29.29	29.00	26.00
Ramachandran[%]§§						
Preferred	91.8	90.9	91.8	91.8	92.2	93.1
Allowed	7.8	8.2	6.5	7.8	7.3	6.5

Outlier	0.4	0.9	1.7	0.4	0.4	0.4
Avr. B-factors[Å^2]						
Protein	44.11	45.38	39.89	39.49	38.64	55.61
Ligand / ion	43.54	49.14	41.94	43.29	42.89	64.04
Water	41.63	44..71	36.69	44.31	39.39	54.30
Wilson B-factor[Å^2]	51.77	50.84	44.09	44.29	42.64	57.06
No. of atoms						
Protein	1836	1836	1896	1816	1845	1845
Ligand / ion	46	46	48	47	46	46
Water	139	139	92	201	70	69
RMSD						
Bond lengths [Å]	0.017	0.077	0.0089	0.0091	0.0094	0.019
Bond Angles [°]	1.777	1.602	1.6500	1.6172	1.6785	1.900
Crystallization condition	3.3 M NaCl, 0.1 M citric acid pH 3.5					
Drop volume [µl]	0.5					
Ratio§§§	3:1:1					
Soak concentration	1.0 M					
Soak duration [min]	10					

Highest resolution shell shown in parenthesis.

§ Relative dose estimated using RADDOSE-3D (Zeldin *et al.*, 2013).

§§ Ramachandran preferred/allowed/outlier regions calculated by Rampage (Lovell *et al.*, 2003).

§§§ Protein to reservoir to water volume ratio.

Table S3 Crystallographic data and refinement statistics.

PDB-ID	*Ls*AA9_A(f) (SSX) 7PXT	*Ls*AA9_A(f) (Ascorbic acid) 7PXU	*Ls*AA9_A(f) (Ascorbic acid) 7PXV	*Ls*AA9_A(f) (RT) 7PXR	*Ls*AA9_A(f) (RT-sync) 7PXS	*Ls*AA9_A(*Ec*) -Cell4 7PXW	*Ls*AA9_A(*Ec*) original structure determination 7PQR
Crystal size [μm^3]	8×10^3	1.5×10^7	1.5×10^7	4×10^6	1.73×10^6	1×10^6	1×10^6
Beamline	ID29 (ESRF)	P11	P11	Diffractometer $	BioMAX (MAXIV)	BioMAX (MAXIV)	BioMAX (MAX IV)
Dose§ [Gy]	7.02×10^4	2.08×10^3	1.70×10^7	-	1.91×10^3	2.14×10^6	-
Wavelength [Å]	0.98	0.97	0.97	1.540	0.9762	0.98	1.127130
Flux [photons/sec]	2.0×10^{10}	5.06×10^8	4.12×10^{12}	-	5.3×10^{10}	5.2×10^{11}	-
Degrees exposed per frame	0.1	0.1	0.1	0.25	0.1	0.1	0.1
Degrees exposed	10	360	360	98	50	360	360
Degrees used in analysis	0-2.5	0-100	0-100	0-98	50	0-360	0-360
Exposure per frame [s]	1	0.03	0.03	10	0.011	0.011	0.011
Space group	$P4_132$	$P4_132$	$P4_132$	$P4_132$	$P4_132$	$P4_1$	$P4_1$
Cell parameters							
(a, b, c)[Å]	125.42	125.02	125.41	126.66	126.63	48.8, 48.9, 109.3	48.92, 48.92, 109.78
(α, β, γ)[°]	90.0	90.0	90.0	90.0	90.0	90.0	90.0
Resolution [Å]	50.0-2.4 (2.46-2.4)	50.0-1.80 (1.85-1.80)	50.0-1.50 (1.54-1.50)	29.85-1.80 (1.84-1.80)	44.77-1.90 (1.95-1.90)	48.0-1.40 (1.44-1.40)	48.9-1.30 (1.33-1.30)
Completeness [%]	88.0 (89.7)	96.9 (99.6)	100.0 (100.0)	100.0 (100.0)	100 (100)	99.6 (99.7)	99.4 (93.3)
R_{meas} [%]	29.3 (91.0)	22.0 (92.1)	16.2 (275.2)	4.7 (50.9)	18.5 (114.7)	11.7 (341.7)	7.7 (125.0)
I/σ(I)	7.21 (3.37)	4.84 (1.11)	16.65 (2.27)	43.8 (4.8)	8.67 (1.37)	12.07 (1.19)	12.2 (1.09)
$CC_{1/2}$ [%]	61.9 (66.4)	98.4 (56.2)	99.9 (59.7)	100.0 (92.5)	99.7 (74.2)	99.9 (55.0)	99.9 (48.2)
Unique reflections	21952 (1698)	30547 (2272)	54321 (3982)	32798 (1930)	27939 (2033)	49868 (3724)	63029 (8661)
Observed reflections	77011 (6103)	130063 (9611)	1113030 (84388)	562827 (19750)	304633 (22850)	678988 (50527)	763391 (26516)
Redundancy	3.51 (3.59)	4.25 (4.23)	20.48 (21.19)	17.2 (10.2)	9.9 (11.24)	13.62 (13.57)	12.1 (5.8)
No. mol./ASU	1	1	1	1	1	1	1
R_{Work} [%]	18.67	19.52	18.84	14.67	16.94	11.71	13.33
R_{Free} [%]	24.76	22.65	20.70	16.62	19.56	15.98	15.33
Ramachandran[%]§§							
Preferred	93.1	94.0	94.4	95.3	94.4	82.7	94.8
Allowed	6.9	6.0	5.6	4.7	5.6	12.3	5.2
Outlier	0.0	0.0	0.0	0.0	0.0	0.5	0.0
Avr. B-factors[Å^2]							
Protein	23.15	17.92	20.13	21.75	27.94	21.5	16.85
Ligand/ion	-	22.95	25.84	44.83	-	35.0	36.36
Water	21.00	27.23	29.13	35.13	34.83	35.4	33.05
Wilson B-factor[Å^2]	36.31	26.55	25.30	27.62	36.19	26.81	22.19
No. of atoms							
Protein	1809	1840	1833	1868	1816	1812	1897
Ligand / ion	0	4	3	13	0	75	24

Water	245	362	269	182	139	302	466
RMSD							
Bond lengths [Å]	0.0073	0.0096	0.0124	0.0141	0.0117	0.0145	0.0142
Bond Angles [°]	1.4641	1.5930	1.7694	1.8438	1.7356	1.8374	1.8243
Crystallization conditions	3.7 M NaCl 0.1 M citric acid pH 4.5	3.5 M NaCl 0.1 M citric acid pH 4.0	3.5 M NaCl 0.1 M citric acid pH 4.0	3.0 M NaCl 0.1 M citric acid pH 3.5	3.0 M NaCl 0.1 M citric acid pH 3.5	2.3 M ammonium sulfate 0.1 M sodium acetate pH 4.2	1.8 M ammonium sulfate 0.1 M sodium acetate pH 4.5
Drop volume [µl]	0.5	2	2	20	4	200	0.3
Ratio§§§	3:1:1	1:1:0	1:1:0	3:1:0	3:1:0	3:1:0	1:1:0
Soaked with	-	Ascorbic acid pH 5.5	Ascorbic acid pH 5.5	-	-	Cellotetraose	-
Soak concentration	-	0.01 M	0.01 M	-	-	0.05 M	-
Soak duration [min]	-	20	20	-	-	30	-

Highest resolution shell shown in parenthesis.

$ In-house diffractometer at the Oak Ridge National Laboratory. Rigaku HighFlux HomeLab instrument with MicroMax-007 HF X-ray generator and Eiger R 4M detector.

§ Relative dose estimated using RADDOSE-3D (Zeldin *et al.*, 2013).

§§ Ramachandran preferred/allowed/outlier regions calculated by Rampage (Lovell *et al.*, 2003).

§§§ Protein to reservoir to water volume ratio.

Table S4 Crystallographic data and refinement statistics for *Ls*AA9_A(*Ec*).

	*Ls*AA9_A(*Ec*)	*Ls*AA9_A(*Ec*)	*Ls*AA9_A(*Ec*)	*Ls*AA9_A(*Ec*)	*Ls*AA9_A(*Ec*)	*Ls*AA9_A(*Ec*)
PDB-ID	7PYL	7PYM	7PYN	7PYO	7PYP	7PYQ
Crystal size [μm^3]	1×10^6	1×10^6	1×10^6	1×10^6	1×10^6	1×10^6
Beamline	P11	P11	P11	P11	P11	P11
Dose§ [Gy]	1.49×10^4	5.61×10^4	2.31×10^5	3.33×10^5	2.13×10^6	6.35×10^6
Wavelength [Å]	1.0332	1.0332	1.0332	1.0332	1.0332	1.0332
Flux [photons/sec]	1.33×10^9	1.33×10^9	1.31×10^{10}	1.31×10^{10}	1.36×10^{11}	1.36×10^{11}
Degrees exposed per frame	0.1	0.1	0.1	0.1	0.1	0.1
Degrees exposed	360	360	720	720	1080	1080
Degrees used in processing	0-80	280-360	0-80	280-360	0-80	280-360
Exposure per frame [s]	0.1	0.1	0.1	0.1	0.1	0.1
Space group	$P4_1$	$P4_1$	$P4_1$	$P4_1$	$P4_1$	$P4_1$
Cell parameters						
(a, b, c)[Å]	48.95, 48.95, 108.99	48.86, 48.86, 108.96	48.93, 48.93, 108.98	48.84, 48.84, 108.90	49.01, 49.01, 109.11	49.19, 49.19, 109.56
(α, β, γ)[°]	90.0, 90.0, 90.0	90.0, 90.0, 90.0	90.0, 90.0, 90.0	90.0, 90.0, 90.0	90.0, 90.0, 90.0	90.0, 90.0, 90.0
Resolution [Å]	50.0-1.70 (1.74-1.70)	50.0-1.75 (1.80-1.75)	50.0-1.40 (1.44-1.40)	50.0-1.40 (1.44-1.40)	50.0-1.60 (1.64-1.60)	50.0-1.60 (1.64-1.60)
Completeness [%]	91.1 (91.3)	94.0 (91.5)	92.2 (88.7)	93.4 (92.7)	90.1 (90.8)	92.6 (94.6)
R_{meas} [%]	14.5 (82.0)	13.0 (64.5)	6.6 (70.6)	5.7 (63.0)	2.7 (10.6)	3.7 (50.4)
I/σ(I)	4.57 (0.87)	4.66 (1.04)	10.0 (1.32)	9.43 (1.25)	22.76 (7.8)	13.37 (1.74)
$CC_{1/2}$ [%]	99.1 (55.4)	99.1 (63.9)	99.9 (72.9)	99.9 (75.4)	99.9 (98.7)	99.9 (76.8)
Unique reflections	50684 (3748)	47775 (3449)	91816 (6541)	92581 (6814)	60403 (4537)	62736 (4780)
Observed reflections	83579 (6112)	77445 (5462)	185902 (13318)	148360 (10744)	99181 (7562)	100497 (7682)
Redundancy	1.65 (1.63)	1.62 (1.58)	2.02 (2.04)	1.60 (1.58)	1.64 (1.67)	1.60 (1.61)
No. mol./ASU	1	1	1	1	1	1
R_{Work} [%]	14.83	16.56	14.23	13.80	12.29	14.42
R_{Free} [%]	18.83	19.39	16.08	16.32	14.66	16.78
Ramachandran[%]§§						
Preferred	94.4	94.4	94.8	94.8	94.8	94.4
Allowed	5.6	5.4	5.2	5.2	5.2	5.6
Outlier	0.0	0.0	0.0	0.0	0.0	0.0
Avr. B-factors[Å^2]						
Protein	17.72	17.95	13.92	14.52	13.01	22.74
Ligand / ion	56.11	53.29	32.64	36.27	34.35	61.02
Water	36.65	26.70	26.72	31.28	27.45	34.11
Wilson B-factor[Å^2]	27.65	27.70	21.72	22.40	22.92	32.41
No. of atoms						
Protein	1807	1795	1806	1832	1810	1831
Ligand / ion	20	20	25	20	20	20
Water	498	260	336	449	350	222
RMSD						

Bond lengths [Å]	0.0088	0.0096	0.0125	0.0124	0.0132	0.0132
Bond Angles [°]	1.4867	1.4772	1.6995	1.6997	1.7650	1.7369

Crystallization condition	2.3 M $(NH_4)_2SO_4$Sodium acetate pH 4.5
Drop volume [µl]	4
Ratio§§§	1:3:0
Oil ratio§§§§	3:2

Highest resolution shell shown in parenthesis.

§ Relative dose estimated using RADDOSE-3D (Zeldin *et al.*, 2013).

§§ Ramachandran preferred/allowed/outlier regions calculated by Rampage (Lovell *et al.*, 2003).

§§§ Protein to reservoir to water volume ratio.

§§§§ Ratio of Paraffin:Silicon oil used for batch crystallization experiment

Table S5 Crystallographic data and refinement statistics for *Ls*AA9_A(*Ec*)-Cell$_3$.

PDB-ID	*Ls*AA9_A(*Ec*)-Cell$_3$ 7PYU	*Ls*AA9_A(*Ec*)-Cell$_3$ 7PYW	*Ls*AA9_A(*Ec*)-Cell$_3$ 7PYX	*Ls*AA9_A(*Ec*)-Cell$_3$ 7PYY	*Ls*AA9_A(*Ec*)-Cell$_3$ 7PYZ	*Ls*AA9_A(*Ec*)-Cell$_3$ 7PZ0
Crystal size [μm^3]	1×10^6	1×10^6	1×10^6	1×10^6	1×10^6	1×10^6
Beamline	P11	P11	P11	P11	P11	P11
Dose§ [Gy]	1.49×10^4	5.62×10^4	2.74×10^5	5.05×10^5	2.97×10^6	9.81×10^6
Wavelength [Å]	1.0332	1.0332	1.0332	1.0332	1.0332	1.0332
Flux [photons/sec]	1.33×10^9	1.33×10^9	1.94×10^{10}	1.94×10^{10}	2.2×10^{12}	2.2×10^{12}
Degrees exposed per frame	0.1	0.1	0.1	0.1	0.1	0.1
Degrees exposed	360	360	720	720	1080	1080
Degrees used in processing	0-80	280-360	0-80	280-360	0-80	280-360
Exposure per frame [s]	0.1	0.1	0.1	0.1	0.1	0.1
Space group	$P4_1$	$P4_1$	$P4_1$	$P4_1$	$P4_1$	$P4_1$
Cell parameters						
(a, b, c)[Å]	48.83, 48.83, 108.78	48.84, 48.84, 108.79	48.84, 48.84, 108.79	48.85, 48.85, 108.79	48.85, 48.85, 108.78	48.90, 48.90, 108.93
(α, β, γ)[°]	90.0, 90.0, 90.0	90.0, 90.0, 90.0	90.0, 90.0, 90.0	90.0, 90.0, 90.0	90.0, 90.0, 90.0	90.0, 90.0, 90.0
Resolution [Å]	50.0-1.40 (1.44-1.40)	50.0-1.40 (1.44-1.40)	50.0-1.60 (1.64-1.60)	50.0-1.20 (1.23-1.20)	50.0-1.6 (1.64-1.60)	50.0-1.2 (1.23-1.20)
Completeness [%]	89.3 (87.4)	96.2 (96.2)	90.1 (89.6)	90.2 (86.8)	90.0 (98.9)	90.6 (88.6)
R_{meas} [%]	10.7 (56.0)	10.7 (58.8)	4.3 (8.7)	5.4 (29.2)	2.4 (3.8)	2.4 (12.4)
I/σ(I)	5.08 (0.98)	5.66 (1.09)	16.68 (8.93)	8.78 (2.10)	33.03 (20.63)	19.53 (4.45)
$CC_{1/2}$ [%]	99.4 (73.2)	99.4 (72.8)	99.7 (98.9)	99.8 (92.4)	99.9 (99.8)	99.9 (98.6)
Unique reflections	88368 (6435)	95196 (7080)	59815 (4435)	141907 (10137)	59808 (4484)	142855 (10325)
Observed reflections	148178 (10664)	204486 (14724)	100546 (7549)	243974 (16845)	100249 (7625)	230046 (15891)
Redundancy	1.68 (1.66)	2.15 (2.08)	1.68 (1.70)	1.72 (1.66)	1.68 (1.70)	1.61 (1.54)
No. mol./ASU	1	1	1	1	1	1
R_{Work} [%]	14.19	13.70	12.59	14..44	12.30	13.40
R_{Free} [%]	16.22	15.34	14.62	15.78	14.38	14.75
Ramachandran[%]§§						
Preferred	94.4	94.4	95.3	94.8	94.8	94.8
Allowed	5.6	5.6	4.7	5.2	5.2	5.2
Outlier	0.0	0.0	0.0	0.0	0.0	0.0
Avr. B-factors[Å^2]						
Protein	13.21	13.33	10.71	12.55	10.74	13.06
Ligand / ion	29.97	30.11	25.39	25.33	25.08	31.60
Water	31.16	31.43	22.38	23.77	22.77	25.81
Wilson B-factor[Å^2]	21.38	21.67	20.28	17.11	20.21	17.44
No. of atoms						
Protein	1904	1904	1825	1825	1825	1839
Ligand / ion	83	83	50	50	50	88
Water	452	452	270	270	269	278
RMSD						
Bond lengths [Å]	0.0129	0.0126	0.0134	0.0150	0.0138	0.0178

Bond Angles [°]	1.8036	1.7881	1.8577	1.8702	1.87	2.0043
Crystallization conditions	2.3 M $(NH_4)_2SO_4$Sodium acetate pH 4.5					
Drop volume [µl]	3					
Ratio§§§	1:2:0					
Oil ratio§§§§	3:2					
Soak concentration	0.5 M					
Soak duration [min]	30					

Highest resolution shell shown in parenthesis.

§ Relative dose estimated using RADDOSE-3D (Zeldin *et al.*, 2013).

§§ Ramachandran preferred/allowed/outlier regions calculated by Rampage (Lovell *et al.*, 2003).

§§§ Protein to reservoir to water volume ratio.

§§§§ Ratio of Paraffin:Silicon oil used for batch crystallization experiment

Table S6 Crystallographic data and refinement statistics for *Ta*AA9_A.

PDB-ID	*Ta*AA9_A 7PZ3	*Ta*AA9_A 7PZ4	*Ta*AA9_A 7PZ5	*Ta*AA9_A 7PZ6	*Ta*AA9_A 7PZ7	*Ta*AA9_A 7PZ8
Crystal size [μm^3]	3.75×10^5	3.75×10^5	3.75×10^5	3.75×10^5	3.75×10^5	3.75×10^5
Beamline	P11	P11	P11	P11	P11	P11
Dose§ [Gy]	5.37×10^3	2.07×10^4	9.56×10^4	2.22×10^5	1.13×10^6	3.12×10^6
Wavelength [Å]	1.0332	1.0332	1.0332	1.0332	1.0332	1.0332
Flux [photons/sec]	1.14×10^8	1.14×10^8	1.11×10^9	1.11×10^9	1.6×10^{10}	1.6×10^{10}
Degrees exposed per frame	0.1	0.1	0.1	0.1	0.1	0.1
Degrees exposed	360	360	720	720	1080	1080
Degrees used in processing	0-120	240-360	0-120	240-360	0-120	240-360
Exposure per frame [s]	0.1	0.1	0.1	0.1	0.1	0.1
Space group	$P2_1$	$P2_1$	$P2_1$	$P2_1$	$P2_1$	$P2_1$
Cell parameters						
(a, b, c)[Å]	34.46, 87.40, 37.47	34.45, 87.37, 37.39	34.41, 87.29, 37.41	34.38, 87.21, 37.33	34.43, 87.38, 37.45	34.41, 87.27, 37.39
(α, β, γ)[°]	90.0, 104.90, 90.0	90.0, 104.96, 90.0	90.0, 104.91, 90.0	90.0, 104.96, 90.0	90.0, 104.89, 90.0	90.0, 104.99, 90.0
Resolution [Å]	50.0-1.90 (1.95-1.90)	50.0-1.85 (1.90-1.85)	50.0-1.45 (1.49-1.45)	50.0-1.45 (1.49-1.45)	50.0-1.80 (1.85-1.80)	50.0-1.40 (1.44-1.40)
Completeness [%]	95.2 (97.4)	88.2 (92.5)	89.6 (83.4)	89.4 (87.2)	85.1 (83.8)	91.1 (91.3)
R_{meas} [%]	23.6 (82.8)	24.8 (96.5)	10.4 (76.7)	10.2 (83.0)	3.8 (9.3)	5.8 (50.9)
I/σ(I)	3.08 (0.82)	2.95 (0.65)	5.71 (1.0)	5.22 (0.82)	16.88 (7.73)	8.83 (1.49)
$CC_{1/2}$ [%]	97.0 (50.4)	97.3 (41.5)	99.5 (51.1)	99.4 (57.9)	99.8 (98.8)	99.8 (74.0)
Unique reflections	16119 (1194)	16153 (1273)	66959 (4641)	66453 (4836)	33301 (2405)	75532 (5685)
Observed reflections	32081 (2349)	34291 (2456)	95287 (6365)	94395 (6335)	43335 (3028)	115762 (8276)
Redundancy	1.99 (1.96)	2.12 (1.93)	1.42 (1.37)	1.42 (1.31)	1.30 (1.26)	1.53 (1.46)
No. mol./ASU	1	1	1	1	1	1
R_{Work} [%]	20.81	23.37	16.56	16.94	13.42	15.12
R_{Free} [%]	25.63	28.58	18.58	18.85	16.98	16.89
Ramachandran[%]§§						
Preferred	96.4	96.4	98.2	97.3	97.8	97.8
Allowed	3.1	3.1	1.3	2.2	1.8	1.8
Outlier	0.4	0.4	0.4	0.4	0.4	0.4
Avr. B-factors[$Å^2$]						
Protein	21.79	21.66	13.95	14.90	11.80	14.51
Water	24.08	18.74	24.38	25.97	23.24	26.99
Wilson B-factor[$Å^2$]	29.34	28.89	21.22	21.76	20.98	21.80
No. of atoms						
Protein	1774	1761	1768	1768	1768	1768
Water	160	63	275	275	275	275
RMSD						
Bond lengths [Å]	0.0089	0.0071	0.0119	0.0113	0.0123	0.0132
Bond Angles [°]	1.7822	1.6659	1.9014	1.8482	1.9179	2.0025

Crystallization conditions	20 mM $MgCl_2$, 0.1 M HEPES pH 7.5, 22 %(w/v) Polyacrylic acid 5100 sodium salt.
Drop volume [µl]	4
Ratio§§§	3:1:0

Highest resolution shell shown in parenthesis.

§ Relative dose estimated using RADDOSE-3D (Zeldin *et al.*, 2013).

§§ Ramachandran preferred/allowed/outlier regions calculated by Rampage (Lovell *et al.*, 2003).

§§§ Protein to reservoir to water volume ratio.

Table S7 Structural parameters of the LPMO Cu-site.

	PDB ID	Res. [Å]	R_{work}/R_{free} [%]	Dose [Gy]	Solvent ligands (Eq, Ax)	Cu-Nδ1 [Å]	Cu-N_{Am} [Å]	Cu-Nε2 [Å]	Cu-O_{Tyr} [Å]	Cu-Eq [Å]	Cu-Ax [Å]	θ_1, θ_2, θ_3 (°)	θ_T (°)	RMSD T (Å)	θ_{H-H} (°)	RMSD H1 (Å)	θ_{H1} (°)	RMSD HN (Å)	θ_{HN} (°)
*Ls*AA9_A(f)	7PXI	1.57	18.30/20.99	7.88×10^3	water, water	1.9	2.2	2.0	2.7	2.2	2.7	92.9, 89.6, 177.1	1.5	0.12	66.9	0.03	5.8	0.02	4.3
	7PXJ	1.75	18.31/21.48	5.99×10^4	water, water	1.8	2.3	2.0	2.7	3.1	3.0	93.1, 90.9, 175.2	2.7	0.07	64.7	0.03	4.7	0.02	3.8
	7PXK	1.40	18.68/20.56	1.39×10^5	water, water	1.9	2.3	2.0	2.8	3.1	3.1	92.0, 95.1, 172.5	2.2	0.04	64.5	0.03	0.9	0.01	8.6
	7PXL	1.30	18.73/19.96	3.59×10^5	water, water	1.9	2.2	2.0	2.8	3.0	3.1	92.1, 96.7, 170.3	4.2	0.04	63.7	0.03	-0.8	0.02	7.4
	7PXM	1.30	18.46/20.23	1.45×10^6	water, water	1.9	2.2	2.0	2.8	3.1	3.2	93.3, 96.6, 169.6	3.1	0.03	63.0	0.03	-3.2	0.01	8.6
	7PXN	1.65	20.35/22.18	6.65×10^6	water, water	1.9	2.3	2.0	2.7	3.3	3.3	91.7, 99.7, 167.5	5.1	0.04	58.6	0.03	-3.2	0.02	7.4
*Ls*AA9_A (*Ec*)	7PYL	1.70	14.83/18.83	1.49×10^4	water, water	2.0	2.1	2.0	2.7	1.9	2.6	91.8, 94.9, 172.6	3.3	0.12	66.2	0.03	0.5	0.02	6.7
	7PYM	1.75	16.56/19.39	5.61×10^4	water, water	2.1	2.2	2.0	2.7	2.0	3.1	92.9, 96.3, 168.6	6.7	0.10	64.3	0.03	-6.1	0.03	6.8
	7PYN	1.40	14.23/16.08	2.31×10^5	water, water	2.0	2.2	2.0	2.8	1.8	3.1	92.6, 97.9, 168.8	4	0.05	65.8	0.03	-6.8	0.02	10.1
	7PYO	1.40	13.80/16.32	3.33×10^5	sulfate, water	1.9	2.3	2.0	2.8	-	3.7	92.3, 99.8, 167.2	4	0.02	64.2	0.03	-9.9	0.03	11.1
	7PYP	1.60	12.29/14.66	2.13×10^6	sulfate, water	2.0	2.3	2.0	2.8	-	3.6	92.7, 99.6, 167.0	4.3	0.03	64.3	0.03	-8.4	0.02	11.5
	7PYQ	1.60	14.42/16.78	6.35×10^6	sulfate, water	2.0	2.2	2.0	2.8	-	3.8	93.3, 102.9, 162.0	7.8	0.05	62.8	0.03	-13.3	0.03	11.3
*Ls*AA9_A(f) (Ascorbic acid)	7PXU	1.80	19.52/22.65	2.08×10^3	water, water	1.9	2.3	2.0	2.9	4.0	3.7	94.8, 96.3, 168.4	3.4	0.02	65.1	0.03	-3.7	0.03	9.7
	7PXV	1.50	18.74/20.70	1.70×10^7	Water, water	1.8	2.3	2.0	2.8	4.0	3.5	93.2, 97.4, 168.6	4.4	0.03	59.3	0.03	-4.9	0.02	9.8
*Ls*AA9_A(f) (SSX)	7PXT	2.40	18.67/24.76	7.02×10^4	water, water	1.9	2.3	1.9	2.7	3.9	2.9	84.9, 99.6, 171.5	7.1	0.10	69.9	0.03	-4.1	0.02	11.1
*Ls*AA9_A(f) (RT)	7PXR	1.80	14.67/16.62	-	water, chloride	1.9	2.2	2.0	2.8	2.2	2.8	88.6, 92.9, 176.6	3.3	0.12	66.3	0.03	5.7	0.03	3.8
*Ls*AA9_A(f) (RT-synchrotron)	7PXS	1.90	16.94/19.56	1.91×10^3	water, water	1.9	2.2	2.0	2.8	2.2	2.6	92.7, 94.7, 170.5	5.9	0.06	63.7	0.02	0.6	0.04	3.5
*Ta*AA9_A	7PZ3	1.90	20.81/25.63	5.37×10^3	water, water	1.9	2.2	2.1	3.0	2.2	2.0	94.0, 91.4, 174.6	-1	0.10	74.7	0.03	2.6	0.03	12.2
	7PZ4	1.85	23.37/28.58	2.07×10^4	water, water	2.0	2.2	2.1	3.0	2.3	2.3	94.4, 89.3, 176.0	-1.5	0.13	82.2	0.03	12.4	0.02	11.6
	7PZ5	1.45	16.56/18.58	9.56×10^4	water, water	1.9	2.2	2.0	3.0	2.1	2.6	93.7, 93.8, 172.5	-0.2	0.08	75.4	0.03	-1.8	0.02	10.1
	7PZ6	1.45	16.94/18.85	2.22×10^5	water, water	1.9	2.2	2.0	3.0	2.3	2.7	94.8, 92.9, 172.3	0.5	0.06	76.9	0.03	-0.6	0.02	10.8
	7PZ7	1.80	13.42/16.98	1.13×10^6	water, water	1.9	2.3	2.0	3.0	2.6	2.8	94.6, 95.2, 170.3	0.5	0.03	75.3	0.04	-3.7	0.02	14.1
	7PZ8	1.40	15.12/16.89	3.12×10^6	water, water	1.9	2.2	2.0	2.9	2.5	3.0	94.6, 96.1, 169.3	1	0.02	74.4	0.03	-4.1	0.02	14.1
*Ls*AA9_A(f)-$Cell_4$	7PYD	2.21	22.47/28.71	7.88×10^3	chloride, NA	2.0	2.3	2.0	2.5	2.3	-	96.4, 94.1, 161.1	15.6	0.08	71.0	0.04	-12.6	0.02	16.1

	7PYE	2.10	23.94/29.42	5.99×10^4	chloride, NA	1.9	2.2	2.0	2.5	2.9	-	95.6, 100.2, 154.1	20.4	0.05	64.3	0.03	-18.1	0.02	17.5
	7PYF	1.90	25.40/28.68	1.39×10^5	chloride, NA	2.0	2.1	2.0	2.5	3.7	-	90.8, 102.0, 152.9	23.5	0.07	55.3	0.03	-20.8	0.02	7.1
	7PYG	1.90	24.29/29.29	3.60×10^5	chloride, NA	2.0	2.1	2.0	2.7	3.8	-	92.9, 103.7, 153.7	20	0.05	60.1	0.03	-20.8	0.02	13.6
	7PYH	1.90	26.15/29.00	1.45×10^6	chloride, NA	1.9	2.3	2.0	2.6	4.0	-	94.8, 107.2, 150.9	18.4	0.16	57.7	0.03	-20.7	0.02	17.9
	7PYI	2.05	21.80/26.00	6.65×10^6	chloride, NA	1.9	2.5	2.0	2.7	3.8	-	98.1, 110.1, 147.9	14.5	0.27	55.4	0.04	-21.7	0.02	24.1
*Ls*AA9_A (*Ec*)-Cell$_3$	7PYU	1.40	14.19/16.22	1.49×10^4	chloride, NA	2.0	2.1	2.0	2.6	2.3	-	91.3, 91.7, 169.2	10.4	0.14	67.7	0.02	-6.2	0.03	4.9
	7PYW	1.40	13.70/15.34	5.62×10^4	chloride, NA	2.0	2.2	2.0	2.6	2.4	-	92.3, 93.6, 169.2	9	0.09	66.4	0.03	-8.3	0.03	8.8
	7PYX	1.60	12.59/14.62	2.74×10^5	chloride, NA	1.9	2.2	2.0	2.7	2.7	-	92.6, 96.8, 167.4	8.3	0.03	65.3	0.02	-8.9	0.03	10.6
	7PYY	1.20	14..44/15.78	5.05×10^5	chloride, NA	1.9	2.3	2.0	2.7	2.8	-	92.5, 97.2, 167.2	8.3	0.01	64.7	0.03	-10.0	0.03	11.2
	7PYZ	1.60	12.30/14.38	2.97×10^6	chloride, NA	1.9	2.3	2.0	2.7	3.6	-	92.8, 98.8, 165.8	8.1	0.02	64.2	0.03	-9.1	0.02	11.0
	7PZ0	1.20	13.40/14.75	9.81×10^6	chloride, NA	1.9	2.3	1.9	2.7	3.8	-	93.0, 98.8, 165.7	8.2	0.03	63.4	0.03	-11.5	0.03	10.0
*Ls*AA9_A (*Ec*)-Cell$_4$	7PXW	1.40	11.71/15.98	2.14×10^6	chloride, NA	2.0	2.3	2.0	2.7	3.9	-	92.4, 99.5, 165.3	8.5	0.04	62.3	0.02	-10.4	0.02	8.0

Table S8 Structural parameters of the LPMO Cu-site.

	PDB ID	Dose [Gy]	Eq-Cu-Ax(°)	N_{Am}-Cu-Eq (°)	N_{Am}-Cu-Ax (°)	N_{d1}-Cu-Eq (°)	N_{d1}-Cu-Ax (°)	N_{e2}-Cu-Eq (°)	N_{e2}-Cu-Ax (°)	O_{Tyr}-Cu-Eq (°)	O_{Tyr}-Cu-Ax (°)	N_{am}-Cu-O_{Tyr} (°)	N_{d1}-Cu-O_{Tyr} (°)	N_{e2}-Cu-O_{Tyr} (°)
*Ls*AA9_A (f)	7PXI	7.88×10^3	94.26	169.77	95.08	91.49	88.62	86.27	89.64	89.23	174.91	81.73	87.58	94.29
	7PXJ	5.99×10^4	98.06	168.28	93.64	88.62	85.28	88.01	91.74	87.74	170.37	80.77	87.19	96.16
	7PXK	1.39×10^5	96.19	169.26	94.46	90.22	85.45	83.2	91.74	90.52	170.02	79.1	87.14	96.4
	7PXL	3.59×10^5	95.19	170.35	94.45	89.44	83.56	82.57	91.58	91.69	168.48	78.86	87.3	98.47
	7PXM	1.45×10^6	97.41	169.6	92.98	87.56	83.7	83.24	92.69	90.74	167.16	78.97	86.76	98.13
	7PXN	6.65×10^6	99.57	168.43	91.98	89.29	81.62	80.54	92.79	88.47	166.33	80.06	87.49	99.46
*Ls*AA9_A (*Ec*)	7PYL	1.49×10^4	93.84	170.53	95.55	87.7	85.83	86.28	90.22	85.92	174.69	84.62	88.86	95.06
	7PYM	5.61×10^4	96.92	163.54	99.47	88.08	81.07	85.04	90.75	79.75	171.55	83.8	91.02	96.66
	7PYN	2.31×10^5	107.18	166.18	86.5	86.74	85.52	84.05	91.13	84.77	166.17	81.41	88.33	97.13
	7PYO	3.33×10^5	NA	NA	96.72	NA	81.74	NA	92.72	NA	168.84	78.44	88.37	92.72
	7PYP	2.13×10^6	NA	NA	96.39	NA	81.28	NA	92.94	NA	169.69	78.33	87.82	98.88
	7PYQ	6.35×10^6	NA	NA	89.43	NA	78.47	NA	93.66	NA	162.71	79.47	88.9	101.67
*Ls*AA9_A (Ascorbic acid)	7PXU	2.08×10^3	NA	NA	89.0	NA	81.3	NA	95.4	NA	161.0	78.6	85.3	100.2
	7PXV	1.70×10^7	NA	NA	89.0	NA	82.9	NA	92.9	NA	163.9	79.5	86.6	99.6
*Ls*AA9_A (SSX)	7PXT	7.02×10^4	NA	NA	96.87	NA	73.96	NA	98.31	NA	164.37	80.4	90.44	97.32
*Ls*AA9_A (RT)	7PXR	-	93.55	172.14	94.22	92.85	87.66	86.07	89.23	91.17	173.93	81.15	88.3	94.9
*Ls*AA9_A (RT-synchrotron)	7PXS	1.91×10^3	94.91	170.19	94.85	89.00	85.67	84.66	87.82	88.86	172.98	81.54	88.48	98.44
*Ta*AA9_A	7PZ3	5.37×10^3	97.91	165.0	96.53	89.36	93	85.2	87.4	82.53	179	83.1	86.1	93.54
	7PZ4	2.07×10^4	102.12	166.92	89.35	91.86	90.24	84.2	91.29	81.66	173.17	87.59	83.92	94.77
	7PZ5	9.56×10^4	108.42	159.05	92.2	84.37	85.77	88.56	94.16	77.22	171.13	81.87	88.03	92.8
	7PZ6	2.22×10^5	103.4	165.93	90.17	89.01	90.19	83.67	89.32	84.36	171.01	82.44	85.36	96.12
	7PZ7	1.13×10^6	104.62	168.2	87.18	86.71	86.7	83.9	93.23	86.08	167.63	82.25	87.76	94.09
	7PZ8	3.12×10^6	105.57	168.13	85.93	89.05	86.97	80.67	92.78	86.03	167.08	82.84	87.57	94.75
*Ls*AA9_A-Cell$_4$	7PYD	7.88×10^3	NA	169.61	NA	88.7	NA	78.68	NA	99.51	NA	89.17	94.44	101.47
	7PYE	5.99×10^4	NA	175.32	NA	86.77	NA	76.23	NA	98	NA	85.95	93.05	108.44
	7PYF	1.39×10^5	NA	171.78	NA	97.1	NA	69.85	NA	96.04	NA	85.35	94.72	109.92
	7PYG	3.60×10^5	NA	170.28	NA	96.45	NA	66.6	NA	97.84	NA	84.02	94.63	107.18

	7PYH	1.45×10^6	NA	NA	NA	NA	NA	NA	NA	NA	NA	81.17	94.95	106.83
	7PYI	6.65×10^6	NA	NA	NA	NA	NA	NA	NA	NA	NA	81.12	93.53	105.32
*Ls*AA9_A (*Ec*)-Cell$_3$	7PYU	1.49×10^4	NA	177.99	NA	90.63	NA	86.44	NA	96.55	NA	82.9	93.66	96.79
	7PYW	5.62×10^4	NA	178.81	NA	88.26	NA	86.02	NA	96.13	NA	82.81	93	96.69
	7PYX	2.74×10^5	NA	175.13	NA	88.13	NA	83.18	NA	94.81	NA	80.36	91.53	98.22
	7PYY	5.05×10^5	NA	176.29	NA	88.99	NA	81.84	NA	95.69	NA	80.87	91.48	98.26
	7PYZ	2.97×10^6	NA	174.62	NA	90.61	NA	78.5	NA	96.32	NA	79.5	90.54	99.56
	7PZ0	9.81×10^6	NA	175.24	NA	89.21	NA	79.74	NA	95.89	NA	79.87	90.62	99.54
*Ls*AA9_A (*Ec*)-Cell$_4$	7PXW	2.14×10^6	NA	171.42	NA	88.07	NA	81.42	NA	90.3	NA	81.14	89.0	101.28

NA: Not applicable, as one atom is not considered coordinating to Cu.

Table S9 Structural parameters of the LPMO Cu-site.

	PDB	Tyr164-OH (σ)	Cu (σ)	O-Cu (Å)	Bond length error	θ_T (°)
*Ls*AA9_A(f) low dose§	5ACG	0.092	0.109	2.72	0.143	2.3
*Ls*AA9_A(f)-Cell$_3$ low dose§	5ACF	0.092	0.105	2.47	0.140	10.10
*Ls*AA9_A(f) low dose	7PXI	0.065	0.068	2.74	0.094	1.5
*Ls*AA9_A(f)-Cell$_4$ low dose	7PYD	0.166	0.221	2.48	0.276	15.6
*Ls*AA9_A(*Ec*) low dose	7PYL	0.072	0.082	2.68	0.109	3.3
*Ls*AA9_A(*Ec*)-Cell$_3$ low dose	7PYU	0.038	0.037	2.55	0.053	10.4
Average (low dose, no substrate)				2.71 ± 0.03		2.37 ± 0.9
Average (low dose, bound substrate)				2.50 ± 0.04		12.03 ± 3.1

§: Published previously (Frandsen *et al.*, 2016)

σ: Single atom diffraction precision index (Kumar *et al.*, 2015).

Bond length error (according to eq 4 in (Gurusaran *et al.*, 2014)).

References

Frandsen, K. E. H., Simmons, T. J., Dupree, P., Poulsen, J. N., Hemsworth, G. R., Ciano, L., Johnston, E. M., Tovborg, M., Johansen, K. S., von Freiesleben, P., Marmuse, L., Fort, S., Cottaz, S., Driguez, H., Henrissat, B., Lenfant, N., Tuna, F., Baldansuren, A., Davies, G. J., Lo Leggio, L. & Walton, P. H. (2016). *Nat. Chem. Biol.* **12**, 298–303.

Gurusaran, M., Shankar, M., Nagarajan, R., Helliwell, J. R. & Sekar, K. (2014). *IUCrJ.* **1**, 74–81.

Kumar, K. S. D., Gurusaran, M., Satheesh, S. N., Radha, P., Pavithra, S., Thulaa Tharshan, K. P. S., Helliwell, J. R. & Sekar, K. (2015). *J. Appl. Crystallogr.* **48**, 939–942.

Lovell, S. C., Davis, I. W., Arendall, W. B., de Bakker, P. I. W., Word, J. M., Prisant, M. G., Richardson, J. S. & Richardson, D. C. (2003). *Proteins Struct. Funct. Bioinforma.* **50**, 437–450.

Tandrup, T., Tryfona, T., Frandsen, K. E. H., Johansen, K. S., Dupree, P. & Lo Leggio, L. (2020). *Biochemistry*. **59**, 3347–3358.

Zeldin, O. B., Gerstel, M. & Garman, E. F. (2013). *J. Appl. Crystallogr.* **46**, 1225–1230.
